# Photon Statistics from Yb^3+^-Doped CsPbCl_3_ Are Inconsistent with Quantum Cutting

**DOI:** 10.1021/acs.nanolett.6c03196

**Published:** 2026-09-25

**Authors:** Vincent R.M. Benning, Faris Horani, Daniel R. Gamelin, Freddy T. Rabouw

**Affiliations:** † Debye Institute for Nanomaterials Science, 8125 Utrecht University, Utrecht, 3584 CC, The Netherlands; ‡ Department of Chemistry, 7284 University of Washington, Seattle, Washington 98195-1700, United States

**Keywords:** photon bunching, Yb^3+^, perovskite, quantum cutting, Auger quenching

## Abstract

CsPb­(Cl_1–*x*_Br_*x*_)_3_:Yb^3+^ has been widely reported as a broadband quantum-cutting material with a photoluminescence quantum yield exceeding 100%, making it a promising candidate for enhancing the blue–green spectral response of silicon photovoltaics. Many groups have reproduced absolute photoluminescence quantum yields over 100%, but others have struggled to obtain such high values. Here, we test the quantum-cutting capabilities of CsPbCl_3_:Yb^3+^ nanocrystals and bulk material using photon-correlation analysis. A quantum-cutting material is expected to exhibit photon bunching, but our experiments on CsPbCl_3_:Yb^3+^ show no such behavior. In fact, we observe the opposite—antibunching—under focused-excitation conditions. This observation can be explained with the previously established Auger-quenching pathway in CsPbCl_3_:Yb^3+^. Our results thus confirm high-power Auger quenching but question earlier descriptions of quantum cutting in CsPb­(Cl_1–*x*_Br_*x*_)_3_:Yb^3+^.

## Graphical abstract

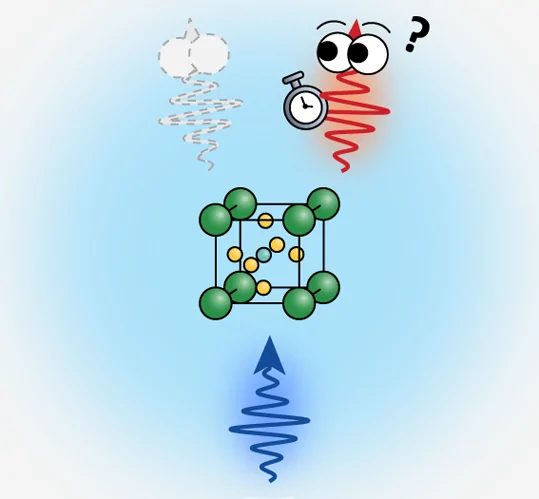

Quantum-cutting phosphors are spectral converters that transform a high-energy photon absorbed into two or more lower-energy photons emitted. The energy efficiency can approach unity, thus avoiding conventional Stokes losses. When integrated with a photovoltaic (PV) cell, such materials can increase the photocurrent by converting high-energy solar photons into multiple lower-energy photons before absorption by the cell.^1–4^

To this end, Yb^3+^ doped CsPbCl_3_ perovskites have attracted particular interest as a promising quantum cutter. These materials combine the strong and broadband absorption of the semiconductor host with the narrow-line emission in the near-infrared (NIR) of Yb^3+^.^5–10^ Moreover, by partial anion exchange of Cl^–^ to Br^–^ the band gap can be tuned to exactly two times the Yb^3+^ excited-state energy.^11,12^ These properties would make the materials ideal for integration with mature silicon-based PV technology.

The quantum-cutting capability of CsPbCl_3_:Yb^3+^ was identified from integrating-sphere measurements revealing photoluminescence quantum yields (PLQYs) exceeding 100%.^5,6,8^ ^,^ ^13–15^ Additionally, spectroscopic data are consistent with a quantum-cutting mechanism involving concerted nonradiative excitation of two Yb^3+^ ions following photoexcitation of the perovskite host, most notably, the strong dependence of the intensity of NIR Yb^3+^ emission on the spectral overlap of the perovskite emission and the absorption associated with double excitation of a pair of Yb^3+^ ions.^16,17^ Furthermore, the observation of a sub-bandgap trap state in CsPbCl_3_ doped with trivalent lanthanides provided a plausible intermediate state for the energy-transfer process from the host exciton to two Yb^3+^ ions.^8,18–21^

At the same time, attempts to reproduce absolute PLQYs approaching 200% have yielded inconsistent results, with reported values frequently below unity.^18,21–29^ Supporting Information Table S1 summarizes the PLQY values reported by various groups. While PLQYs below 100% from integrating-sphere experiments raise questions about the quantum-cutting capabilities of CsPbCl_3_:Yb^3+^, they could also be due to defects in the particular material batch causing nonradiative losses.^30,31^ To resolve this ambiguity, a direct experimental probe for quantum cutting is required.

Here, we employ photon-correlation analysis as an unambiguous test of quantum cutting in Yb^3+^-doped CsPbCl_3_ nanocrystal (NC) ensembles and bulk powders.^32,33^ In a quantum-cutting material, absorption of a single high-energy photon creates two excited states that will emit in short succession. Under continuous-wave illumination this results in an enhanced probability of detecting pairs of photons on the time scale of the emitter excited-state lifetime. However, our photon-correlation analysis reveals no such photon bunching. This observation challenges previous descriptions of quantum cutting in Yb^3+^-doped metal halide perovskites. Under focused excitation, we observe the opposite of photon bunching: photon antibunching. This behavior is consistent with previously studied Auger quenching of the host exciton by excited Yb^3+^ ions.^18,34,35^ This work underpins the importance of photon-statistics-based measurements to interrogate quantum cutting capabilities, and highlights that photon statistics can reveal other nonlinear excited-state processes.

We study two types of CsPbCl_3_:Yb^3+^ samples: one NC sample synthesized via a colloidal method and one microcrystalline powder synthesized via a mechanochemical method (Figure 1a). The nominal Yb^3+^ doping concentrations are 12% for the colloidal sample and 4% for the mechanochemically synthesized sample. SI sections S1.1–S1.8 show the synthesis procedures, preparation and characterization of the samples. Upon excitation of the CsPbCl_3_ host at 375 nm the samples show moderate excitonic emission in the visible around 410 nm and strong NIR emission centered around 980 nm corresponding to the Yb^3+^ ^2^F_5/2_ →^2^F_7/2_ transition (Figure 1b).

**Figure 1:**
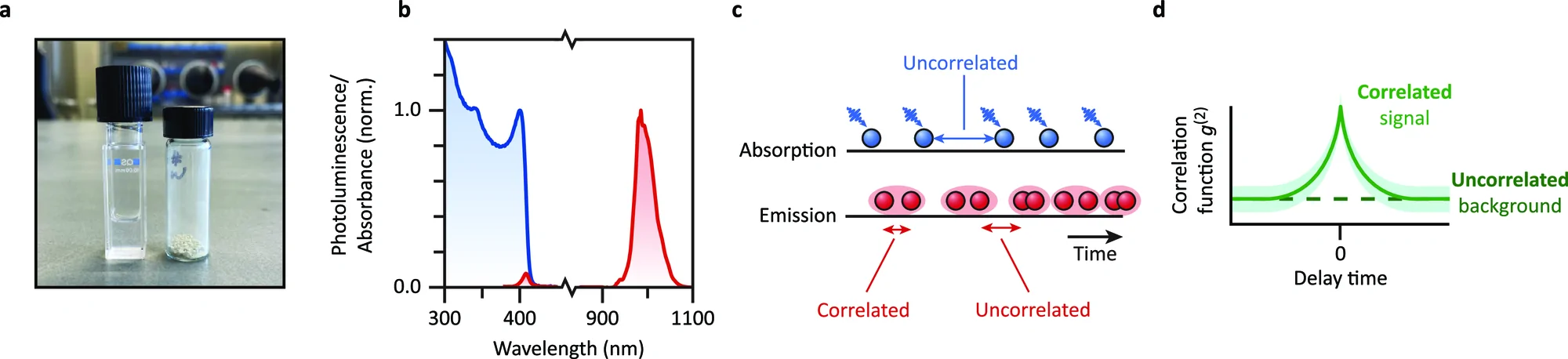
**CsPbCl**_**3**_ **:Yb** ^**3+**^ **samples and photon-correlation concept. (a)** Photograph of CsPbCl_3_:Yb^3+^ synthesized via colloidal methods (nominally 12% Yb^3+^) dispersed in hexane (left) and mechanochemical methods (nominally 4% Yb^3+^) as a microcrystalline powder (right). (b) Representative absorbance spectrum (blue) and photoluminescence spectrum under excitation at λ_exc_ = 375 nm (red). (c) Illustration of hypothetical photon stream from a quantum-cutting material. Continuous-wave excitation produces temporally uncorrelated absorption events. Photon pairs originating from the same absorption event (light red ellipse) are temporally correlated, while photons from different absorption events are not. (d) Schematic illustration of the expected second-order correlation function showing photon bunching. Uncorrelated photons contribute to the flat background (dashed line), whereas correlated photon pairs give rise to a super-Poissonian peak at zero delay time (solid line). The noise on the correlation function is indicated by the teal band.

To test whether the observed NIR emission stems from quantum cutting, we employ photon-correlation analysis.^32,33^ In an ideal quantum-cutting material, absorption of a single high-energy photon results in the emission of two photons. In CsPbCl_3_:Yb^3+^, spectroscopic studies have suggested a mechanism involving concerted excitation of two Yb^3+^ ions.^16,17^ In this scenario, under continuous-wave excitation of an ensemble of emitters, two photons generated following the same absorption event are emitted in short succession and are therefore temporally correlated. This should give rise to a bunching peak centered around zero delay in the second-order correlation function (*g* ^(2)^) (Figure 1c,d). In contrast, emission events originating from different absorption events are uncorrelated in time, resulting in a (nearly) flat background in the *g* ^(2)^. The visibility of the bunching feature is limited by shot noise on the background. In practice, collection losses in the detection setup and nonradiative losses in the sample substantially reduce the detected fraction of correlated photon pairs. Additionally, if one photon of a pair is lost, the remaining photon contributes to the uncorrelated background. Consequently, the background level increases while simultaneously the amplitude of the bunching feature decreases. We carefully optimize and characterize our detection setup (SI section S2). Nevertheless, acquisition times of several tens of hours are required to accumulate sufficient statistics to reliably determine the presence or absence of a bunching feature.

We perform photon-correlation measurements on NC ensembles and microcrystalline aggregates using the automated optical setup shown in Figure 2a. An ultraviolet continuous-wave laser (λ_exc_ = 375 nm) illuminates the sample via a microscope objective, either in wide field or focused. The same objective collects the Yb^3+^ photoluminescence, which is spectrally filtered to reject reflected excitation light and exciton emission using a dichroic mirror and long-pass filter. Subsequently the light is sent to two single-photon avalanche photodetectors (APDs). The *g* ^(2)^ curve is then obtained from the cross-correlation of the photon streams by the two detectors (Figure 3d–g).

**Figure 2:**
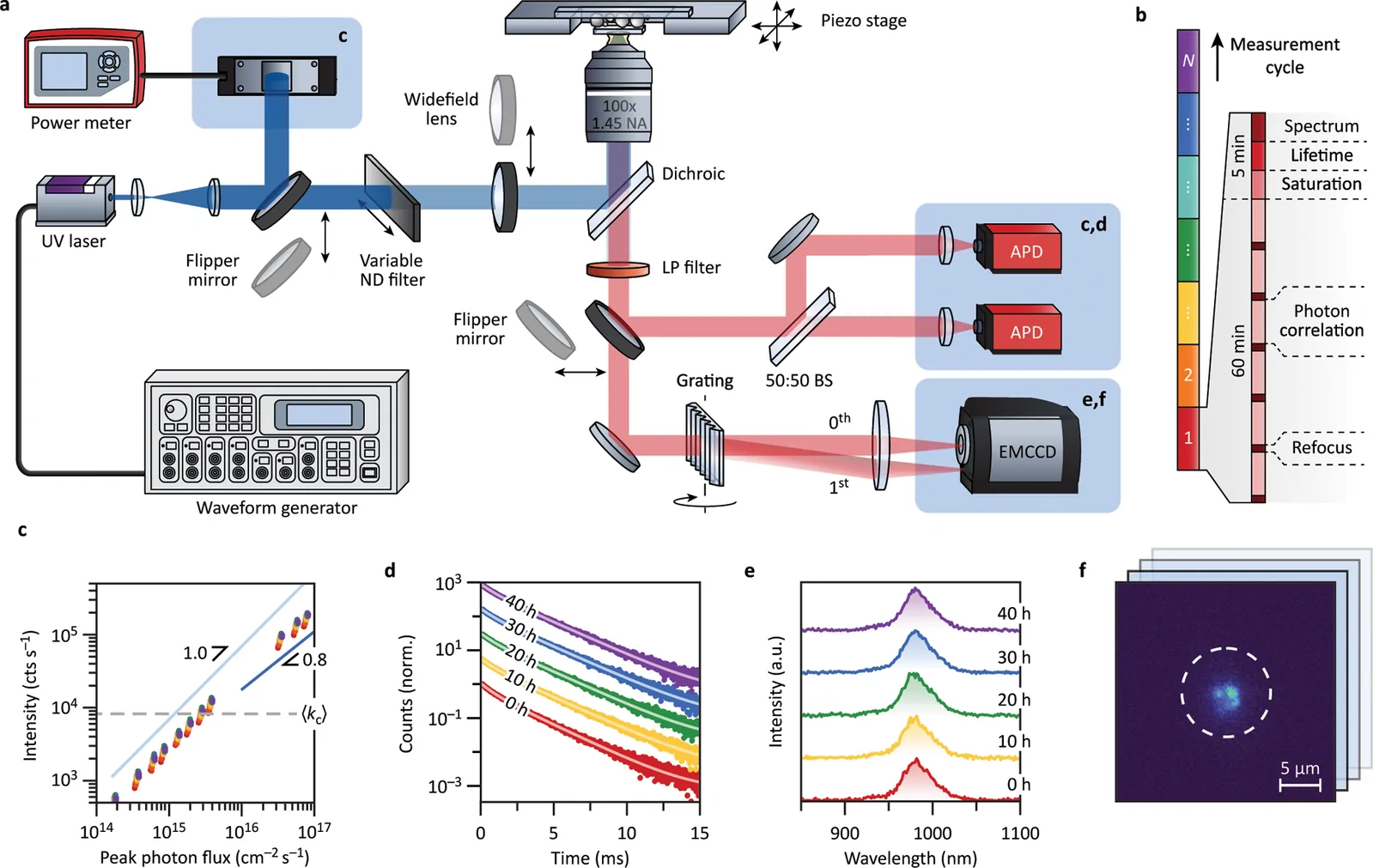
**Automated data-acquisition setup. (a)** Schematic overview of the experimental setup. All optical elements indicated by arrows are motorized and can be rotated or moved into/out of the optical path by the automated measurement routine. Detectors used for the data in panels (c–f) are labeled accordingly. (c) Excitation intensity is measured in the excitation path using a power meter. (c,d) Avalanche photodiodes (APDs) collect Yb^3+^ emission with nanosecond temporal resolution for lifetime and correlation measurements. (e,f) The electron-multiplying CCD camera collects an image of the Yb^3+^ emission for autofocus, or its spectrum if a grating is moved in place. (b) Timeline of the automated measurement routine. (c) Repeated saturation curves (dots), colors from red to purple corresponds to data recorded over 40 h of measurement duration. Solid lines are guides to the eye with power exponents of 1 and 0.8. The dashed line indicates the average count rate used during the photon-bunching experiment, corresponding to an excitation regime well below the onset of the pronounced sublinear response associated with Auger quenching. (d) photoluminescence decay curves (dots) of Yb^3+^ emission (λ_em_ > 900 nm) under excitation at λ_exc_ = 375 nm. (e) Emission spectra of Yb^3+^ at different measurement times. Patterns are offset for clarity. (f) Image of Yb^3+^ photoluminescence of an aggregate of CsPbCl_3_:Yb^3+^ NCs. The white circle shows the projection of the APD pixel onto the sample plane.

**Figure 3:**
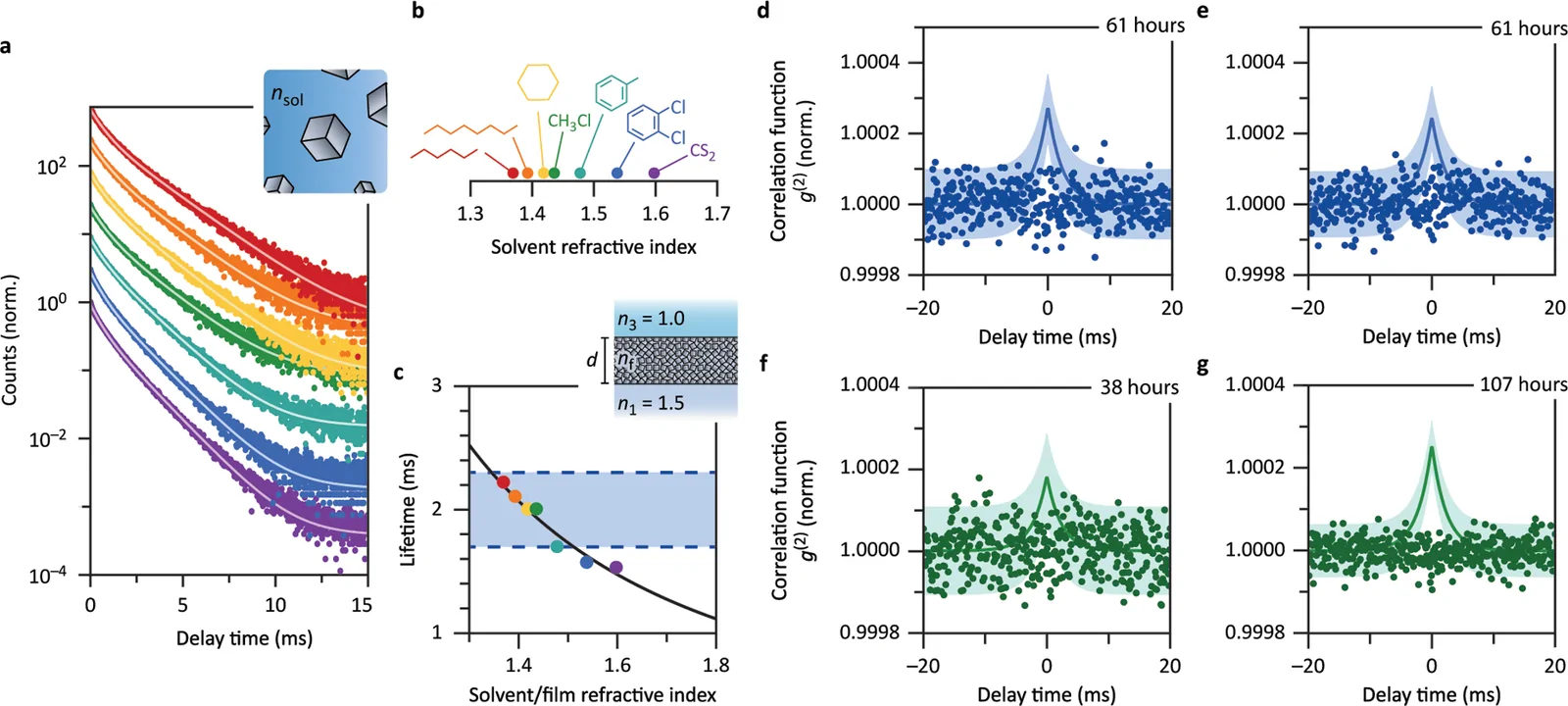
**Dynamics and photon statistics of the Yb** ^3+^ **emission. (a)** Photoluminescence decay curves (dots) of CsPbCl_3_:Yb^3+^ NCs dispersed in solvents of varying refractive index upon excitation at λ_exc_ = 375 nm and recorded at λ_em_ > 900 nm. The solid lines are biexponential fits including a constant background term. Curves are offset for clarity. (b) Refractive indices of solvents used for NC dispersions. (c) Slow lifetime component from the fits in (a) (dots). The black line shows the fit to eq 2. The dark blue dashed lines show the shortest and longest experimental radiative lifetimes measured for the different samples on a glass substrate. **(d–g)** Experimental normalized second-order correlation function (dots) for **(d,e)** two colloidally synthesized samples and **(f,g)** two mechanochemically synthesized samples. Solid lines show the expected correlation function assuming quantum cutting, based on the emission efficiencies and radiative lifetimes from photoluminescence decay experiments. The band indicates the ± 2σ noise on the expected bunching signal. No experimental bunching signal is observed.

An automated measurement routine ensures that the sample remains in focus during the multihour acquisition period and checks sample integrity at regular intervals. To probe the integrity we record hourly photoluminescence saturation curves, time-resolved photoluminescence decay curves, emission spectra, and NIR photoluminescence images (Figure 2b–f). Further details of the optical setup and measurement routine, as well as the full extended data set for all samples, are provided in SI sections S1.6–S1.7 and S2–S3.

The power-dependent measurements (Figure 2c) confirm stable Yb^3+^ emission at the count rates employed in the photon-bunching experiments lying within the approximately linear low-excitation regime and well below the onset of the pronounced sublinear response at higher excitation powers. The observed saturation onset is qualitatively consistent with previous reports, as discussed in SI Section S1.11.^35,36^ Time-resolved Yb^3+^ decay curves recorded at λ_em_ > 900 nm (Figure 2d) are empirically described by a biexponential function including a constant background term. Both lifetime components remain constant throughout the experiment, indicating sample stability. In addition, NIR photoluminescence images and spectra (Figure 2e–f) show no systematic decrease in emission intensity over time, nor any discernible changes in spectral shape or peak position during the measurement, further demonstrating sample stability.

Before we present and analyze the experimental photon correlation, we will calculate the expected bunching magnitude under the assumption that CsPbCl_3_:Yb^3+^ is indeed a quantum cutter. In the absence of significant Auger quenching (SI section S4.7) the expected bunching at τ = 0 is given by

1 $$g^{\left(2\right)}\left(0\right)-1=\frac{k_{r}\eta_{\mathrm{col}}}{2I^{\left(\infty \right)}}$$

where *k*_r_ is the radiative decay rate of the Yb^3+^ excited state, η_col_ is the collection efficiency of our setup (SI section S2) and *I* ^(∞)^ is the steady-state photon count rate (SI section S3).

The bunching amplitude depends directly on *k*_r_. The value of the radiative rate for Yb^3+^ in bulk CsPbCl_3_, *k*_r_^bulk^, is not exactly known. Moreover, for Yb^3+^ in an inhomogeneous optical environment, *k*_r_ may be different from a homogeneous large bulk crystal. This is quantified through the local density of optical states (LDOS), which determines deviations of *k*_r_ from the homogeneous bulk value *k*_r_^bulk^. We will make use of the known scaling of the LDOS with solvent refractive index to determine *k*_r_^bulk^. Next, we will use *k*_r_^bulk^ to estimate *k*_r_ for a film of Yb^3+^-doped NCs or microcrystals lying on a glass substrate.

Specifically, we measure the Yb^3+^ decay dynamics in NC dispersions prepared with solvents of varying refractive indices, *n*_sol_ = 1.38–1.59 (Figure 3a,b). For NCs in a dispersion, the scaling of the LDOS with refractive index—and therefore the scaling of *k*_r_ with refractive index—is known.^37,38^ The Yb^3+^ transition has both magnetic-dipole and electric-dipole character and in general, nonradiative decay is possible.^39,40^ The total decay rate scales as

2 $$k_{\mathrm{Yb}}\left(n_{\mathrm{sol}}\right)=\frac{n_{\mathrm{sol}}^{3}}{n_{\mathrm{NC}}^{3}}k_{\mathrm{MD}}+\frac{n_{\mathrm{sol}}}{n_{\mathrm{NC}}}{\left(\frac{3n_{\mathrm{sol}}^{2}}{2n_{\mathrm{sol}}^{2}+n_{\mathrm{NC}}^{2}}\right)}^{2}k_{\mathrm{ED}}+k_{\mathrm{nr}}$$

where *k*_ED_ and *k*_MD_ are the magnetic- and electric-dipole radiative decay rates in bulk CsPbCl_3_, respectively, and *k*_nr_ is the nonradiative decay rate, which we assume to be solvent-independent.

Figure 3c shows the experimental slow lifetime component as a function of solvent refractive index, revealing a clear dependence consistent with LDOS-induced modification of the radiative decay rate. Fitting eq 2 to this, while keeping *k*_MD_ fixed to the tabulated value of 0.087 ms^–1^,^39^ yields *k*_ED_ = 0.71 ms^–1^ and *k*_nr_ = 0.025 ms^–1^. We consistently use the slow lifetime component in this analysis as a conservative estimate of the radiative contribution; any additional contribution of radiative decay to the faster component would increase the inferred radiative rate and consequently, the expected bunching in the case of quantum cutting.

Using the fitted value of *k*_ED_ and tabulated value of *k*_MD_ we can estimate *k*_r_ for NCs on a glass substrate with air above (Figure 3c). In this three-layer geometry, the LDOS depends on the effective refractive index *n*_eff_ of the NC layer (constituting NCs, ligands, and air gaps), on the local thickness *d* of the NC layer, and on the location of an Yb^3+^ within the NC layer.^41^ Taking reasonable estimates of *n*_eff_ = 1.59 and averaging over *d* = 20 – 100 nm and over emitters positioned at all locations within the layer (SI section S1.5 & S8), we estimate that *k*_r_ = 0.43 ms^–1^. This value is used for *k*_r_ in calculations of the expected bunching amplitude throughout the remainder of this work.^42^ SI section S1.7 explains the three-layer calculation in more detail. The lifetimes measured on NC films approach 1/*k*_r_ to within 0–25% (Figure 3c), indicating quantum yields of Yb^3+^ emission in the film of 75–100%. Due to local variations within the sample composition i.e. layer thickness and distribution of emitters within the slab as well as position-dependent local-field effect in nonspherical NCs, the exact *k*_r_ will vary between NCs, which may contribute to the multiexponential decay observed in Figure 2d and 3a.^43^ Additionally, variations in *k*_nr_ arising for example from differences in defect density or surface environment, may further contribute to the multiexponential decay observed in Figures 2d and 3a. Importantly, the expected bunching amplitude (eq 1) depends directly on *k*_r_, but not on *k*_nr_.

Figure 3d–g show the theoretical prediction of the *g* ^(2)^ for four different samples under the assumption of quantum cutting with the shading showing the expected ± 2σ noise level. The experimentally measured normalized second-order correlation functions for the NC films and microcrystalline samples are overlaid in the panels. In contrast to the predictions, none of the measurements show a bunching feature above the background noise level. Hence, the photon statistics of CsPbCl_3_:Yb^3+^ emission are inconsistent with quantum cutting according to previously proposed mechanisms.

To place this null result in context, we note that the same experimental setup and analysis procedure have previously resolved photon bunching in the established quantum-cutting material YPO_4_:Tb^3+^,Yb^3+^.^32,44^ This confirms that the setup works as expected and the absence of photon bunching from CsPbCl_3_:Yb^3+^ is a property of the material.

Can the absence of photon bunching be reconciled with any previously unconsidered quantum-cutting mechanism in CsPbCl_3_:Yb^3+^? Any pathway that yields two Yb^3+^ emissions within a few ms would produce photon bunching, irrespective of whether the pathway is coherent or incoherent^45^ or whether it is concerted or stepwise transfer.^6,10^ Only a quantum-cutting mechanism that stores part of the excitation energy for times much longer than a few ms could in principle explain our current data. The integrated correlation signal would spread over a wide range of delay times and disappear into the noise background. Such explanation would however produce a slow component with significant amplitude in the photoluminescence decay experiments (Figures 2d,3a). One might also expect different photoluminescence decay curves for host excitation and resonant Yb^3+^ excitation, which we have not yet observed. At present, we cannot think of a quantum-cutting mechanism consistent with both our photon-correlation results and available photoluminescence decay data.

When experiments are performed under focused illumination (Figure 4a) instead of widefield illumination (Figure 3d–g) we observe exactly the opposite of bunching: the emission is antibunched. When focusing the laser, we keep the luminescence count rate constant at approximately 9000 cts/s. A finite number of Yb^3+^ dopants in the excitation volume could in principle produce antibunching. However, we exclude the explanation for our experimental data (Figure 4a) as it predicts a much smaller antibunching amplitude. We estimate that we collect emission of *n*_tot_ ≈ 10^7^ Yb^3+^ ions (Figures 4b, S5–S7). The expected antibunching for *n*_tot_ independent emitters, *g* ^(2)^(0) – 1 = −1/*n*_tot_, would be far below the noise floor and therefore not observable.

**Figure 4:**
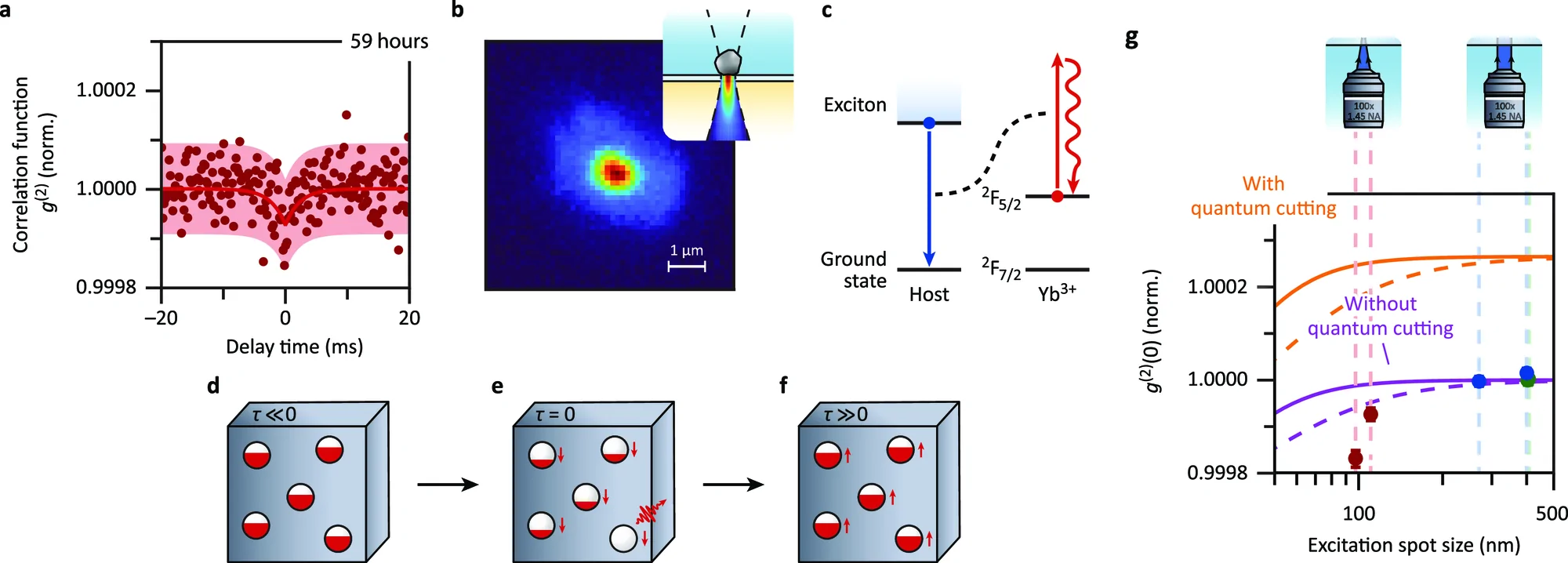
**Antibunching under focused excitation. (a)** Normalized second-order correlation function measured on a CsPbCl_3_:Yb^3+^ microcrystal under focused laser excitation at λ_exc_ = 405 nm. The solid line is a fit to an exponentially decaying antibunching peak with amplitude *g* ^(2)^ – 1 = (−7 ± 1) × 10^–4^. (b) Image of Yb^3+^ photoluminescence of the focused emission spot. Inset shows a schematic of the laser focused on a microcrystal. (c) The Auger quenching process in CsPbCl_3_:Yb^3+^: an exciton in the semiconductor host is transferred to an already excited Yb^3+^, which then nonradiative relaxes back to the emitting state. The host excited-state energy is lost. **(d–f)** Schematic of a volume of CsPbCl_3_ with several Yb^3+^ dopants (circles). The fraction of red filling to the circle depicts the probability that a dopant is in its excited state, explaining the occurrence of antibunching due to the Auger process. The dopants have a steady-state excited-state probability long before (d) or after (f) we detect a photon. The detection of a photon signals that the excited-state probabilities are decreased (e). (g) Experimental photon (anti)­bunching, *g* ^(2)^(0), as a function of excitation spot size. The photon count rate is kept constant between experiments at a target of 9000 cts/s, so experiments with a smaller spot size have a larger local excitation rate density. The corresponding incident excitation powers are provided in Figures S6–S9. Green, blue, and red colors match the correlation data in Figures 3,4. The solid lines are the expected bunching amplitude as a function of spot size if CsPbCl_3_:Yb^3+^ does (orange) or does not (purple) exhibit quantum cutting. The model is explained in detail in SI section S4.

Instead, we interpret the antibunching of *g* ^(2)^(0) – 1 ≈ 10^–4^ as a signature of Auger quenching in Yb^3+^-doped CsPbCl_3_ (Figure 4c), which was previously reported.^35^ The effect of Auger quenching on photon statistics can be understood with a classical model. Conceptually, the model is illustrated in Figure 4d–f. SI sections S4.5–4.9 explain the considerations in detail and derive equations. Briefly, one considers the probabilities that Yb^3+^ dopants are in their excited state with a rate-equation description. At constant excitation, these reach a steady-state value (Figure 4d). The detection of a photon at τ = 0 signals information about the emitting nanocrystal: one of its dopants is now in the ground state. It also changes the excited-state probability of all other dopants. Indeed, just before τ = 0, we know that one dopant was excited. This increases Auger quenching of NC excitons (Figure 4) and thus reduces feeding of all other dopants in the NC. Using the rate constants estimated by Erickson et al.^35^ the result is a decrease in the excited-state probability for all other dopants at τ = 0 (Figure 4e). As τ increases further, the steady state is restored (Figure 4f).

Figure 4g shows the experimental (anti)­bunching *g* ^(2)^(0) as a function of the excitation spot size. Between experiments, we keep the luminescence count rate constant. The expected curve for the scenario *without* quantum cutting (purple) matches the experimental (anti)­bunching. The dependence on spot size is due to Auger quenching, which is affected by the excitation rate density, and by the number of Yb^3+^ dopants in the focal volume (SI section S4.10 for details). An even better match could be obtained if we further optimize the value from the Auger quenching rate or if we assume that some Yb^3+^ ions are optically inactive.^34,46^ Future studies could use photon correlation functions to unravel mechanistic details and quantify parameter values of Auger quenching and other nonlinear processes. In this work, we are satisfied with the qualitative match between experiment and model.

In summary, we have used photon-correlation analysis to directly test the quantum-cutting capabilities of CsPbCl_3_:Yb^3+^ synthesized by mechanochemical and colloidal methods. The theoretically predicted bunching signals, calculated under the assumption of quantum cutting, are inconsistent with the experimentally measured correlation functions for all samples. These results are consistent with reports of <100% photoluminescence quantum yield for CsPbCl_3_:Yb^3+^ and suggest an absence of quantum-cutting behavior in this material. Further work will be required to establish how our findings relate to previous mechanistic studies showing dependence of the NIR Yb^3+^ emission intensity on the bandgap energy of the CsPbCl_3–*x*_Br_*x*_ lattice. At the same time, we observe antibunching of the Yb^3+^ emission under focused excitation. This feature can be understood in terms of previous reported Auger quenching, and highlights the potential of photon correlation analysis to study nonlinear excited-state processes.

## Supplementary Material



## Data Availability

The source data that supports the findings in this work can be downloaded from the Zenodo server (to be added)

## Notes

Additional experimental details on sample fabrication and characterization, experimental methods, detector efficiency, additional data correlation experiments (PDF)
