# Supplementary material for: Photon Statistics from Yb3+-Doped CsPbCl3 Are Inconsistent with Quantum Cutting
Source: Nano Lett. 2026 Sep 25;26(39):13392–8. doi: 10.1021/acs.nanolett.6c03196 (PMC13651601; doi:10.1021/acs.nanolett.6c03196)
Supplement: Supplementary file 1 [file nl6c03196_si_001.pdf]

Photon Statistics from Yb^{3+}-Doped $CsPbCl_3$ are Inconsistent with Quantum Cutting

Vincent R.M. Benning[†], Faris Horani[‡], Daniel R. Gamelin[‡], Freddy T. Rabouw[†,*]

[†] Debye Institute for Nanomaterials Science, Utrecht University, Utrecht, 3584 CC, The Netherlands
[‡] Department of Chemistry, University of Washington, Seattle, Washington 98195-1700, United States
[*] Email: f.t.rabouw@uu.nl

S1 Methods

S1.1 Colloidal Synthesis of Yb^{3+}:$CsPbCl_3$

$CsPbCl_3$:Yb^{3+} nanocrystals were synthesized following a literature procedure reported by Milstein et al.[S1] A mixture of $Pb(OAc)_2 \cdot 3H_2O$ (0.2 mmol), $Yb(OAc)_3$ (0.08 or 0.11 mmol, corresponding to two attempted Yb doping levels), and CsOAc (0.28 mL of a 1 M solution in ethanol) was loaded into a 50 mL round-bottom flask together with 5 mL 1-octadecene, 1 mL oleic acid, and 0.5 mL oleylamine. The reaction mixture was degassed under vacuum at 110 °C for 1 h using a Schlenk line, then placed under N_2 and heated to 240 °C. At this temperature, a solution of 0.2 mL of trimethylsilyl chloride in 0.5 mL 1-octadecene was rapidly injected, and the reaction was immediately quenched by cooling the flask in a room-temperature water bath. After cooling to room temperature, the crude mixture was centrifuged for 15 min at 5000 rpm, and the precipitate was redispersed in hexanes. Nanocrystals were precipitated by addition of ethyl acetate, followed by centrifugation for 10 min at 5000 rpm. The supernatant was discarded, and the product was redispersed in hexanes and centrifuged again for 10 min at 5000 rpm. The final supernatant was filtered through a 0.2 μm PTFE syringe filter. For storage, the nanocrystals were dried and redispersed in anhydrous solvent inside an N_2-filled glovebox.

S1.2 Mechanochemical synthesis of Yb^{3+}:$CsPbCl_3$

$CsPbCl_3$:Yb^{3+} powders were synthesized following a modified literature procedure reported by Crane et al.[S2] CsCl (0.337 g), $PbCl_2$ (0.528 g), and $YbCl_3 \cdot H_2O$ (0.039 g) were loaded into a 50 mL yttria-stabilized zirconia grinding jar charged with approximately 16 g of 6 mm yttria-stabilized zirconia grinding balls. Toluene (10 mL) was added to enable wet grinding. The sealed jar was processed in a planetary ball mill (MSE Supplies; PMGB-0.2L) for 8 h at 500–700 rpm. The resulting white powder was isolated by vacuum filtration, dried, and transferred into a glovebox, where it was annealed at 250 °C for 10 min.

S1.3 Electron Microscopy Characterization

Scanning electron microscopy (SEM) images were acquired using a Phenom Pharos G2 desktop field-emission SEM operating at accelerating voltages of 10—20 kV (Figure S1a,f). Samples were prepared by mounting the powders onto conductive double-sided carbon tape (Electron Microscopy Sciences). Energy-dispersive X-ray spectroscopy (EDX) analysis (Figure S1b—e,g—j) indicates Yb^{3+} incorporation levels of approximately 3.6 and 3.8 at.% relative to the total B-site cations for mechanochemical samples one and two, respectively.

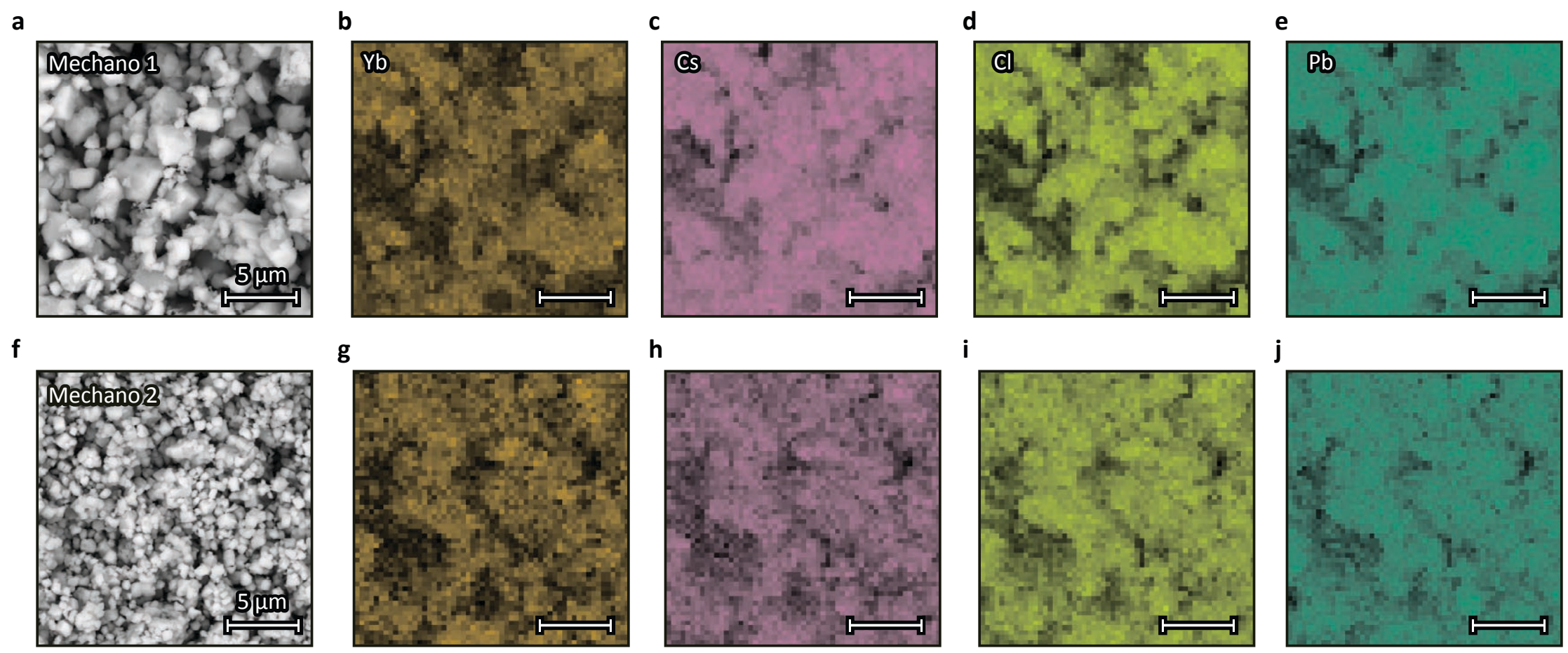

Figure S1: SEM images of mechanochemical sample **(a)** three and **(f)** four. Corresponding elemental distribution maps for Yb, Pb, Cs and Cl are shown for mechnochemical sample **(b–c)** one and **(g–j)** two. The Yb is homogeneously distributed throughout both samples.

Transmission and scanning transmission electron microscopy (TEM/STEM) images were obtained using an FEI Tecnai G2 F20 Supertwin microscope operating at 200 kV. TEM samples were prepared within a glovebox environment by drop-casting NC solutions onto ultrathin carbon Type A 400 Cu grids (TED Pella, Inc.). STEM energy-dispersive X-ray spectroscopy measurements were performed using an EDAX-Elite-T detector.

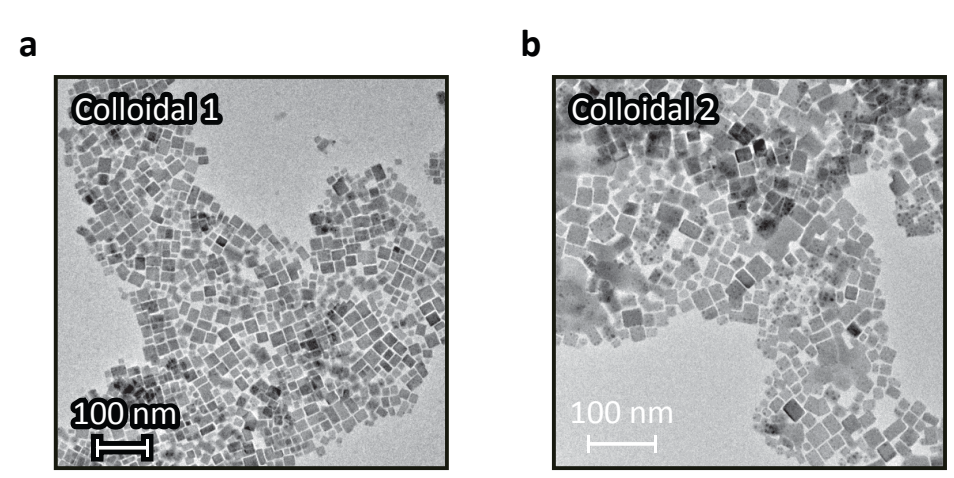

Figure S2: TEM images of colloidal sample **(a)** one and **(b)** two.

S1.4 X-ray diffraction characterization

Benchtop powder XRD patterns were collected using a Bruker D8 Discover diffractometer equipped with a high-efficiency IμS microfocus Cu Kα X-ray source. Scherrer analysis was used to determine

the crystallite sizes for colloidal sample one and two, resulting in an average lateral size of 14 nm for each.

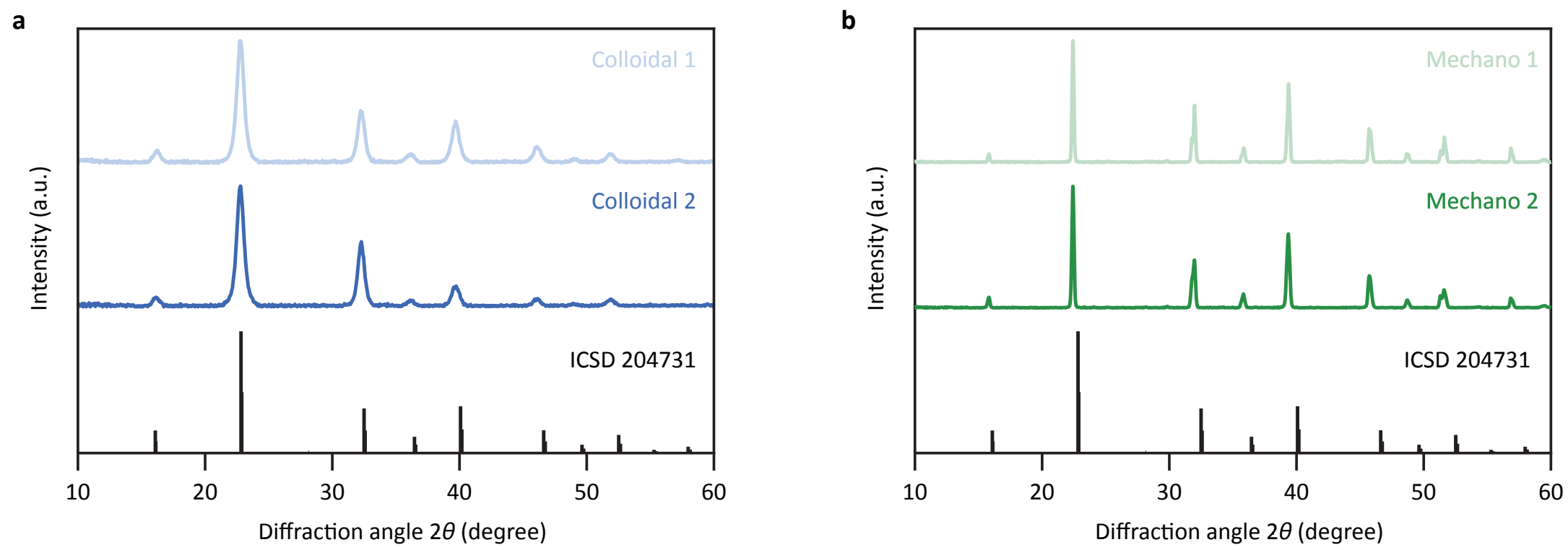

Figure S3: Powder X-ray diffraction pattern of **(a)** colloidally (blue) and **(b)** mechanochemically (green) synthesized $CsPbCl_3$:Yb^{3+} samples. The colloidal samples exhibit broadened diffraction peaks due to their nanoscale crystallite size. The $CsPbCl_3$ reference pattern (black) is indicated by its ICSD collection code. All samples are phase pure, showing no peaks other than those of the reference pattern.

S1.5 Preparation of nanocrystal and microcrystalline samples

Nanocrystal films were prepared using spin coating. The $CsPbCl_3$:Yb^{3+} stock solutions (approximately 10–25 $\mathrm{mg\,mL^{-1}}$) were diluted tenfold in hexane. A 20 μL aliquot of the diluted dispersion was deposited onto a No. 1.5 glass coverslip (Thorlabs CG15CH2) using a Finnpipette and spin-coated at 2500 rpm for 60 s. The sample geometry is shown in Fig. S4a. The thickness of the resulting nanocrystal layer was characterized by atomic force microscopy (JPK NanoWizard II). Prior to AFM imaging, part of the nanocrystal film was removed from the coverslip to expose the underlying glass substrate, which served as a reference plane for determining the film thickness from the height difference between the coated and exposed regions. Although the coverage and height varied across the sample, representative thicknesses were approximately 20–100 nm, corresponding roughly to 1–7 nanocrystal layers, depending on the local coverage. Representative AFM images are shown in Fig. S4d–e.

Microcrystalline samples were prepared from the $CsPbCl_3$:Yb^{3+} powder. Approximately 5 mg of powder was transferred into the well of a Thermo Scientific Gene Frame double-sided adhesive spacer mounted on a microscope slide. A coverslip was then placed over the spacer to enclose the powder. This was followed by gently shaking the assembled sample to distribute the crystallites across the enclosed area. The sample geometry is shown in Fig. S4b.

Optical measurements were performed using an inverted microscope geometry, with the coverslip side of the sample facing the oil-immersion objective. A schematic of the measurement configuration and the sample position relative to the objective is shown in Fig. S4c.

S1.6 Single-photon time correlation experiments

Time-correlated single-photon counting measurements were performed using a home-built microscope setup. The Yb^{3+}-doped $CsPbCl_3$ colloidal samples were prepared by spin-coating 25 μL of a

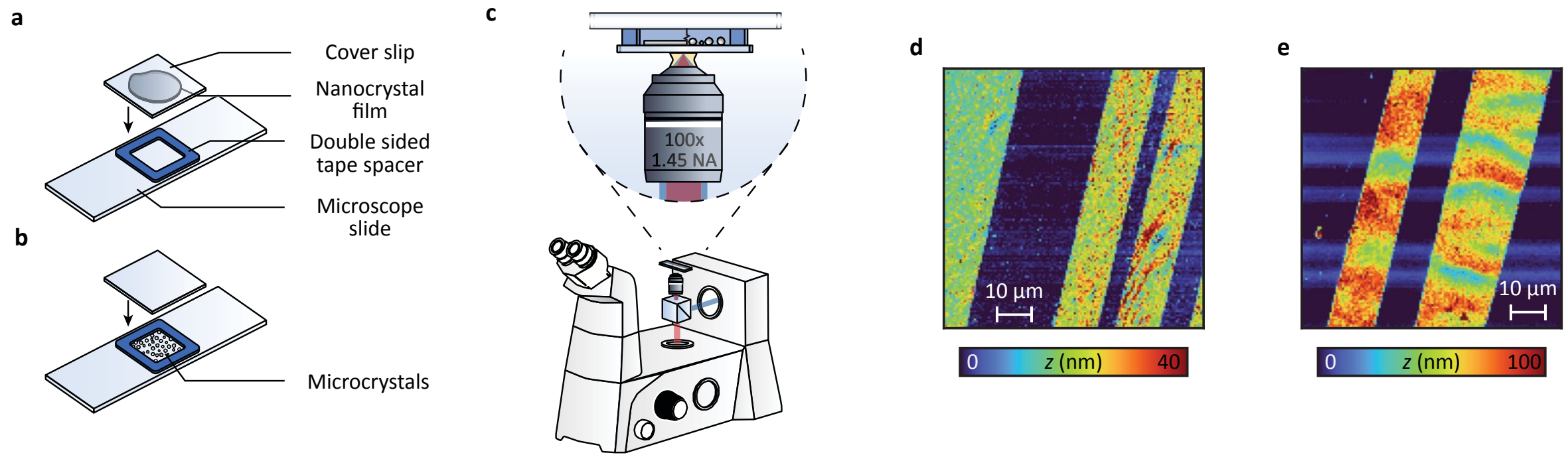

Figure S4: (a) Geometry of the spin-coated nanocrystal sample. **(b)** Geometry of the enclosed microcrystalline powder sample. **(c)** Schematic of the inverted-microscope configuration used for the optical measurements, showing the sample position relative to the oil-immersion objective. **(d–e)** Representative atomic force microscopy images of a spin-coated nanocrystal sample. Blue regions indicate exposed glass used as the height reference.

100× diluted stock solution onto a coverslip at 2500 rpm. The mechanochemical samples were not further processed prior to deposition on the coverslip.

For both widefield and focused measurements, the samples were excited using an OBIS 375LX laser and a PicoQuant LDH-D-C-405 laser operated in continuous-wave mode. The laser power was controlled by inserting neutral density filters in the excitation path using two motorized filter wheels (Thorlabs FW102C loaded with FW102CWNEB and FW212CWNEB, respectively). The excitation light was focused onto the sample using a Nikon CFI Plan Apochromat Lambda D 100× oil-immersion objective.

The sample emission was collected by the same objective and imaged with a 19-mm lens (Thorlabs AC127-019-B-ML) onto two avalanche photodiodes (Thorlabs SPDMH2), arranged in a Hanbury-Brown–Twiss configuration using a 50:50 beamsplitter plate (Thorlabs BSW11R). Photon detection events from the avalanche photodiodes were registered by a time tagger (quTools quTAG MC), resulting in a photon stream with a temporal resolution of 1 ps. Correlations in this photon stream were analyzed and corrected for detector artifacts (dead time, afterpulsing, and afterglow) using the strategies detailed in our previous work.[S3]. In brief, afterglow and afterpulsing events were removed from the photon stream by applying a time gate that excludes all detection events within a 50 ns window following each detection event. To isolate the near-infrared (NIR) emission from reflected excitation light and visible excitonic emission, a 650-nm dichroic mirror (Thorlabs DMLP650R) and a 900-nm long-pass filter (Thorlabs FELH0900) were placed in the detection path.

To correct for axial drift during the correlation experiment an autofocus algorithm was run every 10 minutes. A piezo stage (Mad City Labs Nano-LPS300) was used to scan along the z-axis, after which the sample was repositioned to the z-coordinate corresponding to optimal focus. Images were acquired using an EMCCD camera (Andor iXon Ultra 888). For correlation experiments under focused excitation, a 1000 mm focal length lens (Thorlabs LA1464) was inserted into the excitation path via a motorized flipper mirror prior to scanning to change from focused to widefield illumination (Thorlabs MFF101). Lateral drift was corrected using a phase-correlation algorithm to determine positional offsets, after which the sample was returned to the reference position recorded at the start of the experiment.

S1.7 Spectroscopic measurements

Photoluminescence emission spectra were recorded using the same excitation source and detector as used for drift correction. The emitted light was directed into a spectrograph (Andor Kymera) for spectral dispersion.

Photoluminescence lifetime measurements were performed on the same setup used for time-correlated single-photon counting measurements. Yb^{3+} ions were excited using an OBIS 375LX laser operated in pulsed mode. The laser was driven by an Aim-TTi TGA1244 waveform generator, producing pulses with a repetition rate of 67 Hz and a pulse width of 100 μs. Absorption spectra of diluted nanocrystal solutions were recorded using an Agilent Cary 60 spectrophotometer.

S1.8 Effective refractive index of the nanocrystal film

The effective refractive index of the nanocrystal film was estimated using a volume-weighted average of the refractive indices of the nanocrystal and oleic-acid ligand. A nanocrystal edge length of $l_{\mathrm{NC}} = 14$ nm (Sec. S1.4), an oleic-acid ligand length of $l_{\mathrm{OA}} = 1.9$ nm, and refractive indices $n_{\mathrm{NC}} = 1.8823$ and $n_{\mathrm{OA}} = 1.4661$ were used.[S4–S6] The nanocrystal volume fraction within the ligand-coated particle is:

$$\phi_{\mathrm{NC}} = \frac{l_{\mathrm{NC}}^3}{(l_{\mathrm{NC}} + 2l_{\mathrm{OA}})^3} = 0.487 \tag{S1}$$

The refractive index of the ligand-coated nanocrystal is then:

$$n_{\mathrm{coat}} = \phi_{\mathrm{NC}} n_{\mathrm{NC}} + (1 - \phi_{\mathrm{NC}})\, n_{\mathrm{OA}} = 1.67 \tag{S2}$$

Because the packing density of the nanocrystals within the film is not known precisely, two limiting geometries were considered. For close-packed spheres, using a packing fraction $\phi_{\mathrm{cp}} = \pi/(3\sqrt{2})$, the remaining volume was assumed to be air with $n = 1$. This gives:

$$n_{\mathrm{eff,cp}} = \phi_{\mathrm{cp}} n_{\mathrm{coat}} + (1 - \phi_{\mathrm{cp}}) = 1.50 \tag{S3}$$

For the limiting case of a fully space-filled film:

$$n_{\mathrm{eff,sf}} = n_{\mathrm{coat}} = 1.67 \tag{S4}$$

For the subsequent analysis, the mean of these two limiting values was used:

$$n_{\mathrm{eff}} = \frac{n_{\mathrm{eff,cp}} + n_{\mathrm{eff,sf}}}{2} = 1.59 \tag{S5}$$

S1.9 Calculating k_{r} for $CsPbCl_3$:Yb^{3+} on a glass substrate

Using the parameter values for k_{ED} and k_{MD} from the systematic experiments in different solvents of Fig. 3 in the main text, we estimate k_{r} for our sample on a glass substrate with the method of Karaveli and Zia.[S7] We consider a three-layer geometry with glass (refractive index $n = 1.5$), $CsPbCl_3$:Yb^{3+} (assuming $n_{\mathrm{eff}} = 1.59$ due to a combination of semiconductor, air, organics), and air ($n = 1$). We calculate the acceleration or slow down of electric-dipole and magnetic-dipole transition rates compared to k_{ED} and k_{MD} using eq 2 of Karaveli and Zia.[S7] The result is averaged over different thicknesses of the $CsPbCl_3$:Yb^{3+} layer between 20 and 100 nm and over all position within the $CsPbCl_3$:Yb^{3+} layer. This yields the estimated value of $k_{\mathrm{r}} = 0.43$ ms^{-1}.

S1.10 Conversion of incident optical power to peak photon flux

The photon flux incident on the samples was determined from the excitation power measured before the light passed through the neutral-density (ND) filter wheels and microscope optics (Fig. 2a). At this position, the optical power is highest, minimizing the relative uncertainty of the power measurement. The optical power reaching the sample was subsequently obtained by correcting the measured power for the wavelength-dependent transmission of the ND filters and for transmission losses in the microscope optics, which were determined independently.

The optical power reaching the sample is

$$P_{\text{sample}} = P_{\text{meas}} T_{\text{ND}} T_{\text{opt}} \tag{S6}$$

where P_{meas} is the power measured before the ND filters and microscope, T_{ND} is the transmission of the ND-filters, and T_{opt} is the transmission of the microscope optics.

The spatial intensity distribution of the excitation beam at the sample was approximated by a normalized two-dimensional Gaussian,

$$I(x, y) = \frac{P_{\text{sample}}}{2\pi\sigma^2} \exp\left[-\frac{x^2 + y^2}{2\sigma^2}\right] \tag{S7}$$

where σ_{spot} is the Gaussian width of the excitation spot. This definition is normalized such that integration over the complete beam profile yields the total optical power,

$$\int_{-\infty}^{\infty} \int_{-\infty}^{\infty} I(x, y)\, \mathrm{d}x\, \mathrm{d}y = P_{\text{sample}} \tag{S8}$$

At the center of the excitation spot, the intensity therefore reaches its maximum value,

$$I_{\text{peak}} = \frac{P_{\text{sample}}}{2\pi\sigma^2} \tag{S9}$$

The corresponding peak photon flux is obtained by dividing the peak intensity by the photon energy,

$$\Phi_{\text{peak}} = \frac{I_{\text{peak}}}{E_{\text{ph}}} \tag{S10}$$

where

$$E_{\text{ph}} = \frac{hc}{\lambda_{\text{exc}}} \tag{S11}$$

S1.11 Comparison of Auger quenching onset with literature

To compare the excitation regime in our experiment to the saturation behavior reported by Erickson et al., we estimate the peak excitation rate per nanocrystal from:

$$k_{\text{exc}} = \sigma_{\text{abs}} \Phi_{\text{peak}} \tag{S12}$$

where Φ_{peak} is the incident photon flux (section S1.10) and σ_{abs} is the absorption cross section of $CsPbCl_3$ at 375 nm. We use an absorption cross section of 4.3×10^{-14} cm^2 at 365 nm reported by Erickson et al. The transition to sublinear Yb^{3+} emission (Figure 2c of the main text; panel d of Figure S6–S9) then occurs at peak excitation rates on the order of 300 s^{-1}, with some variation

between samples. This value is approximately a factor of 3 lower than the 1000 s^{-1} reported by Erickson et al. for pronounced excitation-induced quenching, but is comparable to the saturation regime reported by Sokolova et al. of $\sim$300 s^{-1}. (after converting the excitation photon flux in the paper to an excitation rate).[S8;S9] The variation between reported saturation regimes may reflect differences in doping concentration, excitation profile, particle size and morphology or sample geometry and the resulting local electromagnetic-field intensities.

S2 Spectral detection efficiency

The wavelength-dependent collection efficiency was determined from the ratio of the intensity of a spectrally filtered (LLTF Contrast) white light laser (SuperK FIU-15) measured at the sample position to that measured at the detector position. The intensity at the sample position was measured using a power meter and subsequently converted to an incident photon flux using:

$$\Phi_{\text{laser}}(\lambda) = \frac{\lambda P_{\text{laser}}(\lambda)}{hc} \tag{S13}$$

where $P_{\text{laser}}(\lambda)$ is the measured optical power at the wavelength λ, h is Planck's constant, and c is the speed of light. For the detector count rate measurements, the power meter was replaced by a silver mirror to redirect the light towards the detectors. To avoid detector saturation, neutral density filters were placed in the detection path during the measurement of the detector count rates. The wavelength-dependent transmission of these filters, $T_{\text{ND}}(\lambda)$, was taken into account. The collection efficiency for each detector as a function of wavelength (Figure S4a) was then obtained via:

$$\eta(\lambda) = \frac{k_{\text{det}}(\lambda)}{\Phi_{\text{laser}}(\lambda) T_{\text{ND}}(\lambda)} \tag{S14}$$

where $k_{\text{det}}(\lambda)$ is the measured count rate at the detector position. The effective collection efficiency was subsequently determined by weighting the wavelength-dependent collection efficiency with the normalized Yb^{3+} emission spectrum, $S(\lambda)$, such that $\sum_i S(\lambda_i) = 1$:

$$\eta_{\text{eff}} = \sum_i \eta(\lambda_i) S(\lambda_i) \tag{S15}$$

Using this approach, effective collection efficiencies of 0.5% and 0.6% were obtained for the two detectors (Figure S4b).

The efficiencies obtained from this calibration quantify the transmission and detection efficiency of the optical setup. For sample emission, an additional uncertainty arises from the fraction of emitted light that enters the objective, which depends on the sample geometry and radiation pattern. For our NA = 1.45 objective and relevant refractive indices, a simple isotropic-emission estimate gives a collected fraction of approximately 20–30%. Since the emitters are located close to dielectric interfaces, the actual radiation pattern can deviate substantially from isotropic emission, with preferential coupling toward the higher-index side of the interface. Therefore, the actual collected fraction may plausibly approach 50%. We thus estimate an uncertainty of approximately a factor of two in the effective collection efficiency. Because the predicted bunching amplitude scales linearly with collection efficiency, this translates directly into an approximately factor-of-two uncertainty in the predicted bunching amplitude. Even at the lower bound of this estimate, the predicted bunching remains resolvable above the experimental noise. Our estimate of k_{r} is conservative because it is derived from the slowest decay component from Fig. 3a. If the faster component also contains

a radiative contribution, the true k_r would be larger, increasing the predicted bunching amplitude. Thus, uncertainty in k_r is likely to act opposite to a possible reduction from a lower collection efficiency.

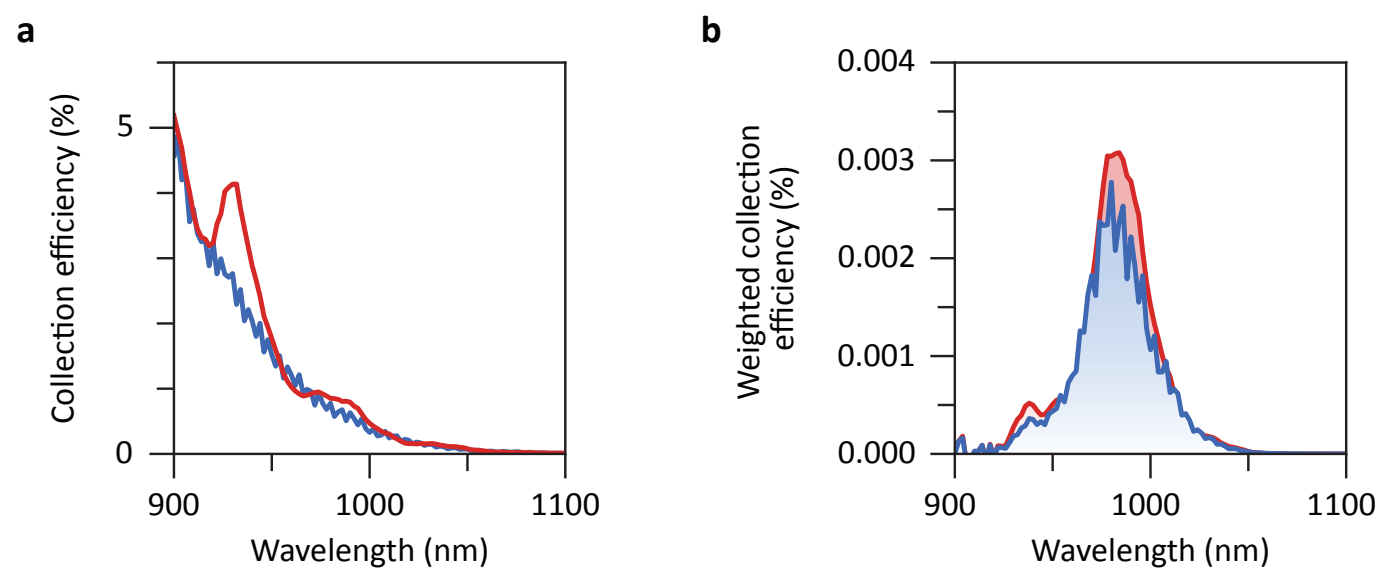

Figure S5: (a) Wavelength-depedent collection efficiency of avalanche photodiode one (blue) and two (red). **(b)** Wavelength dependent collection efficiency weighted using the normalized NIR emission spectrum of $CsPbCl_3$:Yb^{3+} from Figure 1b

S3 Extended data

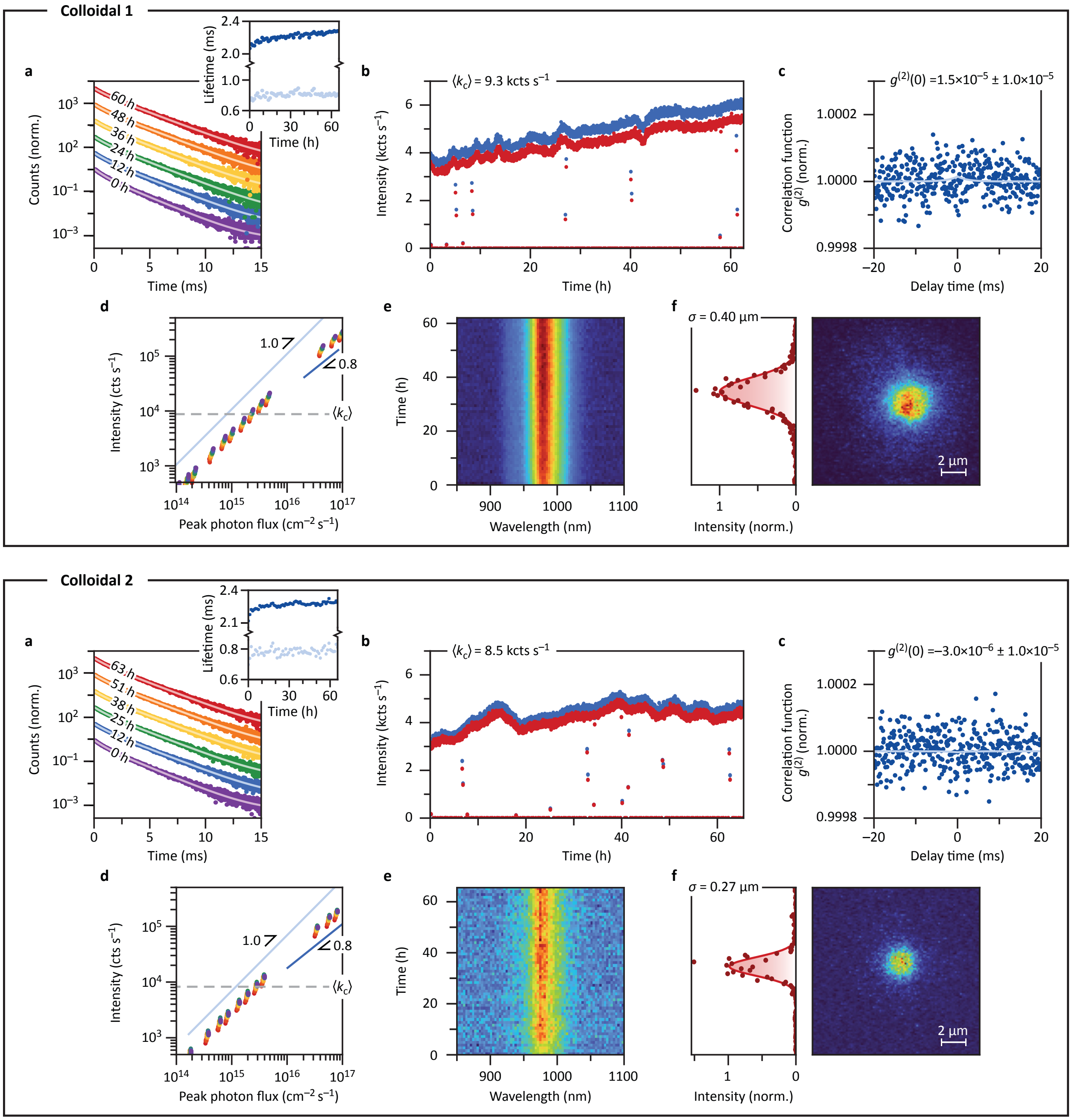

Figure S6

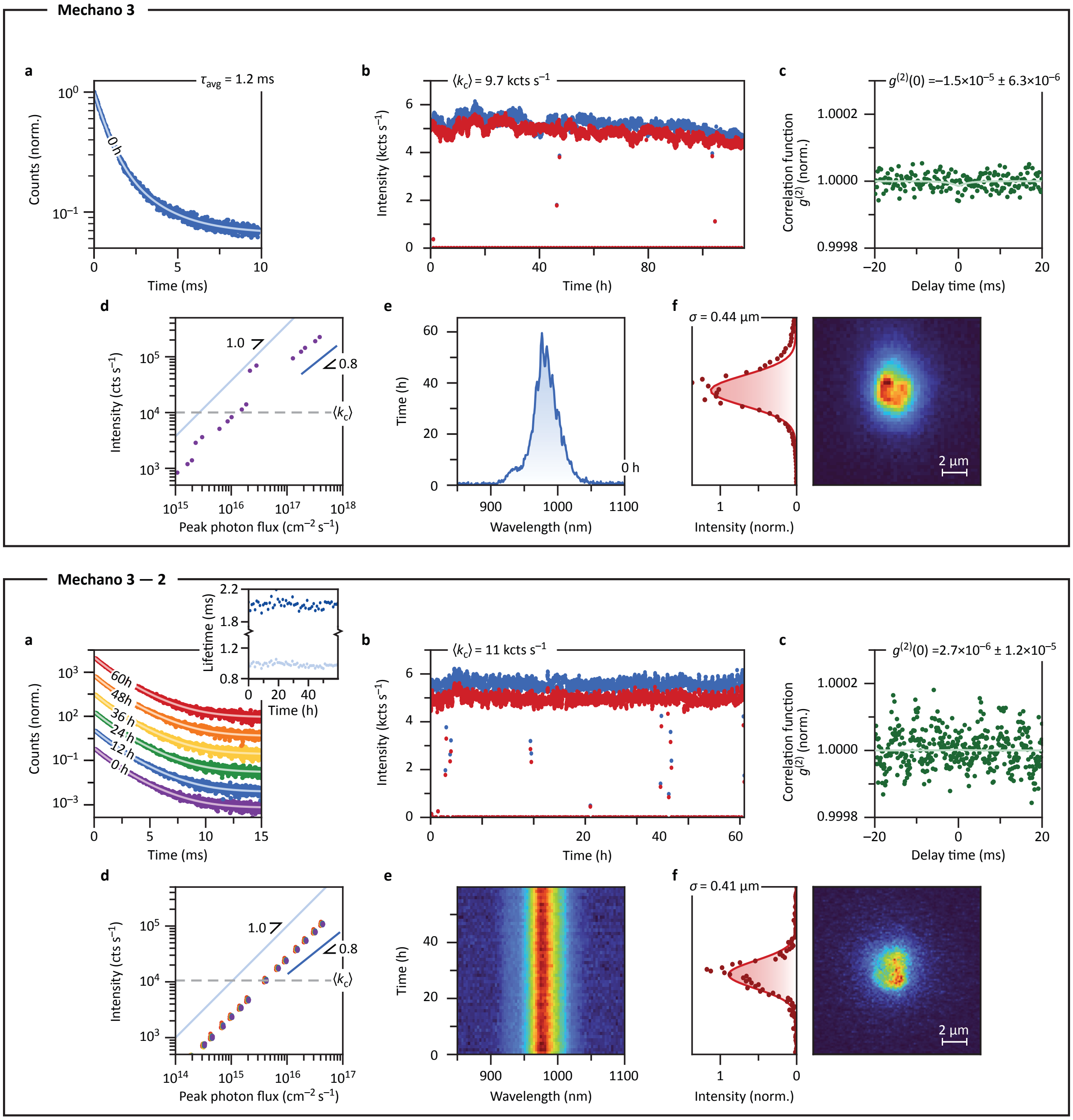
Mechano 3
a
τ_{avg} = 1.2 ms
Counts (norm.)
0 h
Time (ms)
b
$\langle k_c \rangle$ = 9.7 kcts s^{-1}
Intensity (kcts s^{-1})
Time (h)
c
$g^{(2)}(0)$ = −1.5×10^{-5} ± 6.3×10^{-6}
Correlation function $g^{(2)}$ (norm.)
Delay time (ms)
d
Intensity (cts s^{-1})
1.0
0.8
$\langle k_c \rangle$
Peak photon flux (cm^{-2} s^{-1})
e
Time (h)
0 h
Wavelength (nm)
f
σ = 0.44 µm
Intensity (norm.)
2 µm
Mechano 3 — 2
a
Lifetime (ms)
Time (h)
60h
48h
36 h
24 h
12 h
0 h
Counts (norm.)
Time (ms)
b
$\langle k_c \rangle$ = 11 kcts s^{-1}
Intensity (kcts s^{-1})
Time (h)
c
$g^{(2)}(0)$ = 2.7×10^{-6} ± 1.2×10^{-5}
Correlation function $g^{(2)}$ (norm.)
Delay time (ms)
d
Intensity (cts s^{-1})
1.0
0.8
$\langle k_c \rangle$
Peak photon flux (cm^{-2} s^{-1})
e
Time (h)
Wavelength (nm)
f
σ = 0.41 µm
Intensity (norm.)
2 µm

Figure S7

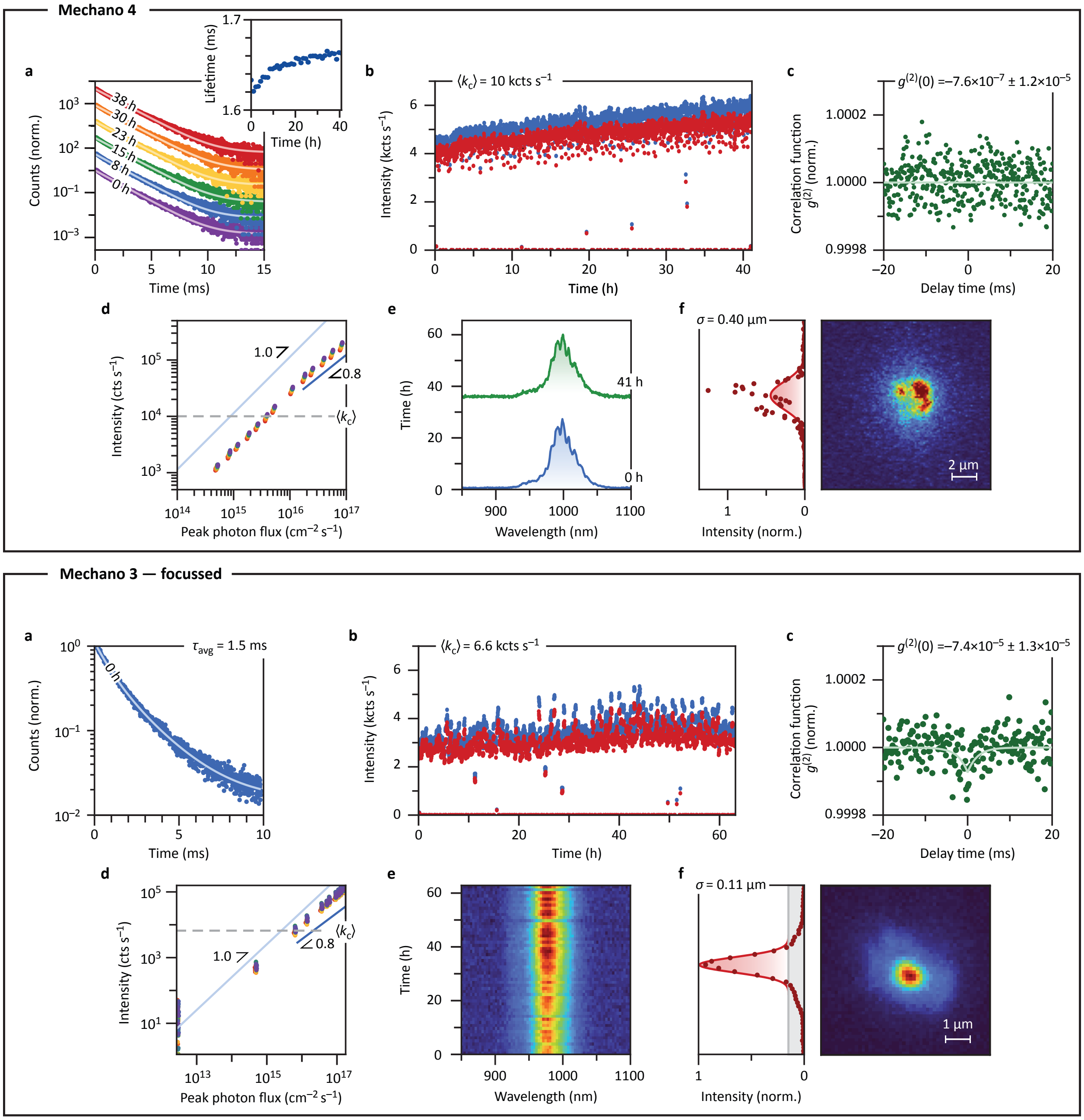

Mechano 4
a
Lifetime (ms)
Time (h)
38 h
30 h
23 h
15 h
8 h
0 h
Counts (norm.)
Time (ms)
b
⟨k_c⟩ = 10 kcts s^−1
Intensity (kcts s^−1)
Time (h)
c
g^(2)(0) = −7.6×10^−7 ± 1.2×10^−5
Correlation function g^(2) (norm.)
Delay time (ms)
d
Intensity (cts s^−1)
1.0
0.8
⟨k_c⟩
Peak photon flux (cm^−2 s^−1)
e
Time (h)
41 h
0 h
Wavelength (nm)
f
σ = 0.40 µm
Intensity (norm.)
2 µm
Mechano 3 — focussed
a
τ_avg = 1.5 ms
0 h
Counts (norm.)
Time (ms)
b
⟨k_c⟩ = 6.6 kcts s^−1
Intensity (kcts s^−1)
Time (h)
c
g^(2)(0) = −7.4×10^−5 ± 1.3×10^−5
Correlation function g^(2) (norm.)
Delay time (ms)
d
Intensity (cts s^−1)
1.0
0.8
⟨k_c⟩
Peak photon flux (cm^−2 s^−1)
e
Time (h)
Wavelength (nm)
f
σ = 0.11 µm
Intensity (norm.)
1 µm

Figure S8

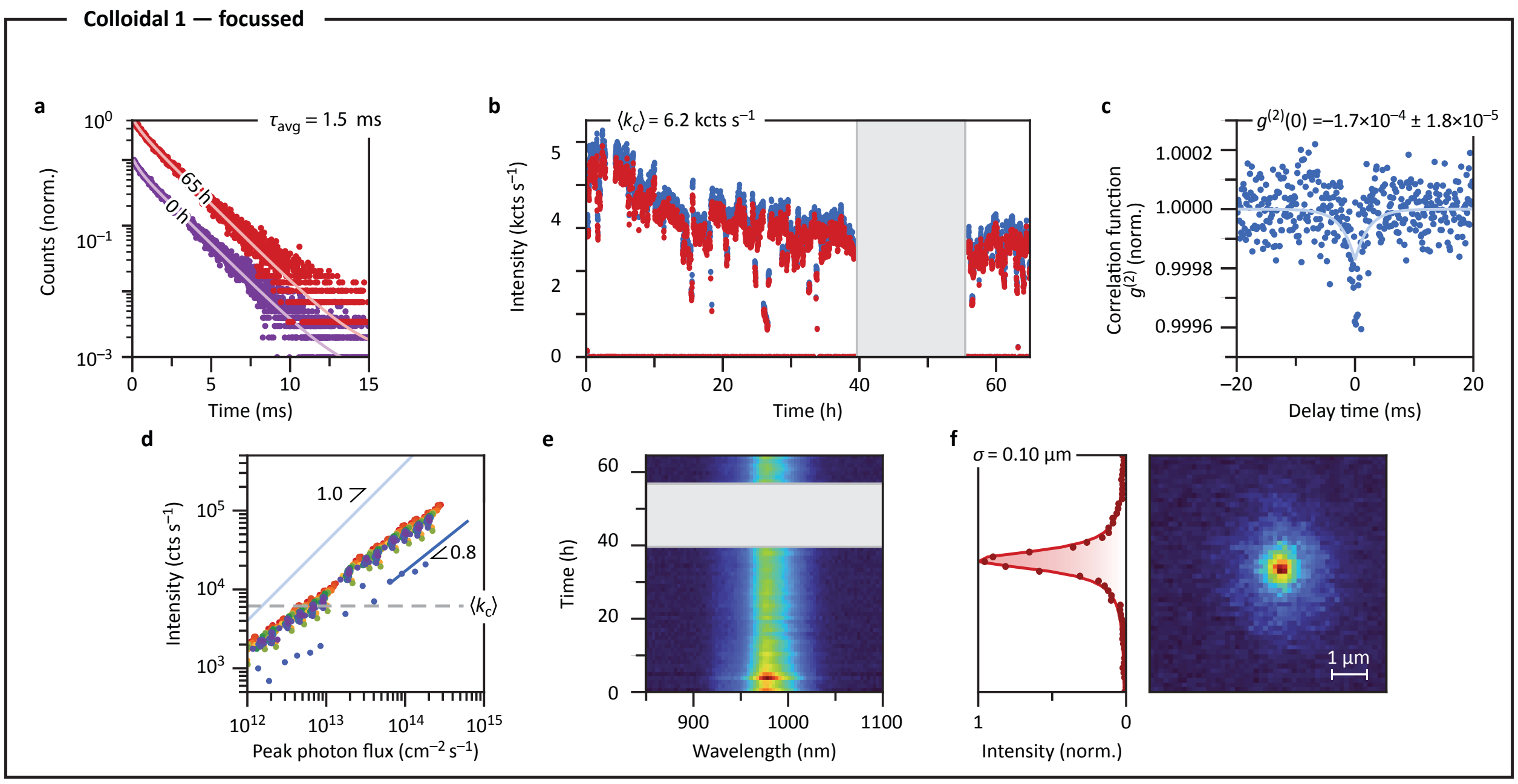

Figure S9: Extended characterization during photon-correlation measurements. Figures S6–S9 show the data recorded during the photon-correlation measurements. **(a)** Repeated Yb^{3+} photoluminescence decay curves acquired over the course of the experiment, with time progressing from purple to red. Insets show the fitted lifetimes of the fast and slow decay components as a function of measurement time. **(b)** Intensity traces recorded on APD1 and APD2. The count rates fluctuate between measurement cycles, which we attribute mainly to the finite precision of the autofocus routine in returning to the same sample position. **(c)** Normalized second-order correlation function of the Yb^{3+} emission. The solid line shows a fit in which the decay rate is fixed to the estimated radiative decay rate, $k_r = 0.43\ ms^{-1}$, and the amplitude is used to quantify the degree of bunching or antibunching. **(d)** Repeated saturation curves recorded during the experiment. Solid lines indicate power-law slopes of 1 and 0.8 and serve as guides to the eye for assessing the onset of saturation. The dashed gray line indicates the average count rate used during the correlation measurement. **(e)** Repeated Yb^{3+} emission spectra, shown as heat maps where multiple spectra were available and as individual spectra where only one or two spectra were recorded. Variations in spectral intensity coincide with the count-rate fluctuations in **(b)**, whereas the spectral shape remains unchanged. **(f)** Fluorescence image of the Yb^{3+} emission spot. The side panel shows a cross section through the center of the spot, with the data shown as dark-red dots and the solid line representing a Gaussian fit. For some datasets, fewer reference measurements were collected.

S4 Bunching and anti-bunching for emitters fed from a common host material

S4.1 Strategy

To calculate the expected normalized photon correlation function at zero time delay, $g^{(2)}(0)$, for continuous-wave excitation of a luminescent sample we must evaluate:

(A) The steady-state count rate $I^{(\infty)}$.

(B) The expected count rate directly following the detection of a photon $I^{(0)}$.

We then calculate

$$g^{(2)}(0) = \frac{I^{(0)}}{I^{(\infty)}} \tag{S16}$$

S4.2 Independent emitters

First consider a sample of n independent two-level emitters, each absorbing at a rate k_{exc} and emitting at a total decay rate k_{Yb}, which is the sum of a radiative contribution k_{r} and a nonradiative contribution k_{nr}. For each emitter separately, the probability p_1 that it is excited evolves with time as

$$\frac{\mathrm{d}p_1}{\mathrm{d}t} = k_{\text{exc}}p_0 - k_{\text{Yb}}p_1, \tag{S17}$$

where $p_0 = 1 - p_1$ is the probability that it is in the ground state. The steady-state solution $p_1^{(\infty)}$ is reached when

$$k_{\text{exc}}\left(1 - p_1^{(\infty)}\right) - k_{\text{Yb}}p_1^{(\infty)} = 0. \tag{S18}$$

The solution to this,

$$p_1^{(\infty)} = \frac{k_{\text{exc}}}{k_{\text{exc}} + k_{\text{Yb}}}, \tag{S19}$$

determines the steady-state count rate due to the combined signal of each n emitters:

$$I^{(\infty)} = \eta_{\text{col}}k_{\text{r}}np_1^{(\infty)}, \tag{S20}$$

where $\eta_{\text{col}}k_{\text{r}}$ is a constant.

Directly after the detection of a photon, we know that one of the emitters is in its ground for certain ($p_1 = 0$). We have no information about any of the other $n-1$ emitters, so they still have an excited-state probability of $p_1 = p_1^{(\infty)}$. Hence, the expected count rate is

$$I^{(0)} = \eta_{\text{col}}k_{\text{r}}(n-1)p_1^{(\infty)}. \tag{S21}$$

For the normalized photon correlation function at zero time delay, we obtain the well known relation

$$g^{(2)}(0) = 1 - \frac{1}{n} \tag{S22}$$

S4.3 Emitters populated by rapid 1-to-1 energy transfer from the host material; microstate model

Now consider one nanocrystal containing n dopant emitters that are populated by energy transfer. We assume that a host nanocrystal absorbs at a constant rate k_{exc} and transfers its energy to one of the dopants that is presently un-excited on a much faster time scale than any other decay process. The total rate at which the number of excited emitters increases is always k_{exc}, determined by the absorption by the host nanocrystal. Here, we first consider the limit in which host-to-dopant energy transfer occurs without competing quenching pathways.

We now construct a coupled-rate-equation model that describes the probabilities p_i that exactly i of the n emitters are excited:

$$\frac{\mathrm{d}p_{i+1}}{\mathrm{d}t} = k_{\mathrm{exc}} p_i - (i+1)\, k_{\mathrm{Yb}} p_{i+1}. \tag{S23}$$

This equation describes that if each emitter decays with a rate constant of k_{Yb}, the total rate of decay from the microstate with $i+1$ excited emitters to the one with i excited emitters is $(i+1)\, k_{\mathrm{Yb}}$. This description yields a recursive equation for the steady-state situation (i.e., $\mathrm{d}p_i/\mathrm{d}t = 0$ for all i). We obtain the steady-state solution

$$p_i^{(\infty)} = \frac{A}{i!} \left(\frac{k_{\mathrm{exc}}}{k_{\mathrm{Yb}}} \right)^i. \tag{S24}$$

In the scenario that $k_{\mathrm{exc}}/k_{\mathrm{Yb}} \ll n$, the populations decrease sufficiently quickly for increasing i and the normalization constant A becomes an exponential such that

$$p_i^{(\infty)} = \frac{\mathrm{e}^{-k_{\mathrm{exc}}/k_{\mathrm{Yb}}}}{i!} \left(\frac{k_{\mathrm{exc}}}{k_{\mathrm{Yb}}} \right)^i. \tag{S25}$$

We recognize a Poisson distribution of microstate populations with mean $\mu = k_{\mathrm{exc}}/k_{\mathrm{Yb}}$. This yields a steady-state count rate of

$$I^{(\infty)} = \eta_{\mathrm{col}} k_{\mathrm{r}} \sum_{i=0}^{n} i\, p_i^{(\infty)} = \eta_{\mathrm{col}} k_{\mathrm{r}} \frac{k_{\mathrm{exc}}}{k_{\mathrm{Yb}}}. \tag{S26}$$

If we detect a photon, the probability π_i that it was emitted by microstate i is proportional to the expected emission rate $i\, p_i^{(\infty)}$ of that microstate. The normalized probability π_i is

$$\pi_i = \frac{i\, p_i^{(\infty)}}{\sum_{j=0}^{n} j\, p_j^{(\infty)}}. \tag{S27}$$

Just after the detection of the photon emitted by microstate i, the system must be in microstate $i-1$. Hence, the probability that the system is in microstate $i-1$ just following the detection of a photon is

$$p_{i-1}^{(0)} = \pi_i. \tag{S28}$$

Hence, the expected count rate just following the detection of a photon is

$$I^{(0)} = \eta_{\rm col} k_{\rm r} \sum_{i=0}^{n} (i-1)\, p_{i-1}^{(0)} = \eta_{\rm col} k_{\rm r} \frac{\sum_{i=0}^{n} i(i-1)\, p_i^{(\infty)}}{\sum_{j=0}^{n} j\, p_j^{(\infty)}} =$$
$$\eta_{\rm col} k_{\rm r} \frac{\sum_{i=0}^{n} i^2\, p_i^{(\infty)} - \sum_{i=0}^{n} i\, p_i^{(\infty)}}{\sum_{j=0}^{n} j\, p_j^{(\infty)}} = \eta_{\rm col} k_{\rm r} \left[\frac{\sum_{i=0}^{n} i^2\, p_i^{(\infty)}}{\sum_{j=0}^{n} j\, p_j^{(\infty)}} - 1 \right] \quad \text{(S29)}$$

We see that the last expression divides the second moment of the Poisson distribution ($\mu^2 + \mu$) by the mean (μ). We obtain

$$I^{(0)} = \eta_{\rm col} k_{\rm r} \left[\frac{(k_{\rm exc}/k_{\rm Yb})^2 + k_{\rm exc}/k_{\rm Yb}}{k_{\rm exc}/k_{\rm Yb}} - 1 \right] = \eta_{\rm col} k_{\rm r} \frac{k_{\rm exc}}{k_{\rm Yb}}. \quad \text{(S30)}$$

$g^{(2)}(0)$ for this scenario is

$$g^{(2)}(0) = 1. \quad \text{(S31)}$$

S4.4 Emitters populated by rapid 1-to-1 energy transfer from the host material; self-consistent excited population

We can describe the situation above in terms of the excited-state population of each separate emitter, although they are coupled. The excited-state population of each, p_1, follows

$$\frac{\mathrm{d}p_1}{\mathrm{d}t} = \frac{k_{\rm exc}}{n p_0}\, p_0 - k_{\rm Yb} p_1. \quad \text{(S32)}$$

Here, $p_0 = 1 - p_1$ is the probability for each emitter to be in its ground state. As the host nanocrystal must distribute its absorbed energy over np_0 ground-state dopants, each of them has an effective excitation rate of

$$k_{\rm exc}^{\rm eff}(n) = \frac{k_{\rm exc}}{n p_0}. \quad \text{(S33)}$$

This expression uses a single time-averaged np_0 to define an effective excitation rate, while neglecting fluctuations of the number of ground-state dopants. This is an acceptable approximation of a nanocrystal with $n = 800$ dopants, but may not be valid for n of order 1. The steady-state population $p_1^{(\infty)}$ is the one for which $\mathrm{d}p_1/\mathrm{d}t = 0$, which is

$$p_1^{(\infty)} = \frac{k_{\rm exc}}{n k_{\rm Yb}} \quad \text{(S34)}$$

and a steady-state count rate of

$$I^{(\infty)} = \eta_{\rm col} k_{\rm r} n p_1^{(\infty)} = \eta_{\rm col} k_{\rm r} \frac{k_{\rm exc}}{k_{\rm Yb}}. \quad \text{(S35)}$$

This is the same expression as in the previous section.

In this model, the detection of a photon at $t = 0$ provides information about the 1 emitter that emitted the photon. We know that this 1 emitter is in its ground state at $t = 0$ and was in its excited state just before $t = 0$. It also provides information about the other $n - 1$ emitters. We know that, just before $t = 0$, these emitters were excited more strongly by a small amount. Indeed,

just before $t = 0$, the host material divided its absorbed energy not over np_0 but over $(n-1)p_0$ ground-state dopants. After all, 1 of the dopants was surely excited just before $t = 0$ so it could not accept more absorbed energy. Just before $t = 0$, the populations (p_0 and p_1) of the $n-1$ other dopants followed

$$\frac{\mathrm{d}p_1}{\mathrm{d}t} = \frac{k_{\mathrm{exc}}}{(n-1)p_0}\, p_0 - k_{\mathrm{Yb}} p_1. \tag{S36}$$

Solving for the steady-state situation, $\mathrm{d}p_1/\mathrm{d}t = 0$, among the $n-1$ other dopants, we obtain their excited-state population at $t = 0$:

$$p_1^{(0)} = \frac{1}{n-1} \frac{k_{\mathrm{exc}}}{k_{\mathrm{Yb}}} \tag{S37}$$

We predict a count rate just after the detection of a photon of

$$I^{(0)} = \eta_{\mathrm{col}} k_{\mathrm{r}} (n-1) p_1^{(0)} = \eta_{\mathrm{col}} k_{\mathrm{r}} \frac{k_{\mathrm{exc}}}{k_{\mathrm{Yb}}}. \tag{S38}$$

and a $g^{(2)}(0)$ of

$$g^{(2)}(0) = 1. \tag{S39}$$

Although this section does not explicitly account for discrete excited-state populations and fluctuations therein, this result is the same as in the previous section. This section provides intuition for why $g^{(2)}(0) = 1$: the detection of a photon signals that 1 dopant is in the ground state, but the other dopants have a slightly increased probability to be in their excited state. The reason is the more effective transfer of energy to any of the other dopants just before the photon was detected.

S4.5 Emitters populated by 1-to-1 energy transfer from the host material; the host material is quenched by an Auger process; microstate model

Now we again consider one nanocrystal doped with n emitters. Now energy transfer of a host excitation is no longer transferred to the dopants at infinite speed, but instead there is competition with an Auger process. An excitation of the host material can be transferred to any of the dopants at a rate $(n-i)k_{\mathrm{ET}}$, where i is the number of already excited dopants. The excited dopants will quench the host excitation at a rate $i\, k_{\mathrm{Auger}}$. The coupled-rate-equation model becomes

$$\frac{\mathrm{d}p_{i+1}}{\mathrm{d}t} = k_{\mathrm{exc}} \frac{(n-i)k_{\mathrm{ET}}}{(n-i)k_{\mathrm{ET}} + i\, k_{\mathrm{Auger}}} p_i - (i+1) k_{\mathrm{Yb}} p_{i+1}, \tag{S40}$$

where the fraction describes the efficiency of energy transfer due to competition with Auger quenching. The solution for the steady state is

$$p_i^{(\infty)} = A \frac{n!}{(n-i)!\, i!} \left(\frac{k_{\mathrm{exc}}}{k_{\mathrm{Yb}}}\right)^i \left(\frac{k_{\mathrm{ET}}}{k_{\mathrm{ET}} - k_{\mathrm{Auger}}}\right)^i \Gamma\!\left(\frac{n k_{\mathrm{ET}}}{k_{\mathrm{ET}} - k_{\mathrm{Auger}}} - i + 1\right). \tag{S41}$$

Here $\Gamma(x)$ is the gamma function. For the case of no quenching ($k_{\mathrm{Auger}} = 0$), the gamma function goes to $(n-i)!$. We thereby retrieve the expression for $p_i^{(\infty)}$ of section S4.3 as expected.

Following the same arguments as in section S4.3, we can express the steady-state count rate

and the expected count rate just after the detection of a photon.

$$I^{(\infty)} = \eta_{\text{col}} k_{\text{r}} \, \frac{\sum_{i=0}^{n} i \, p_i^{(\infty)}}{\sum_{i=0}^{n} p_i^{(\infty)}} \tag{S42}$$

$$I^{(0)} = \eta_{\text{col}} k_{\text{r}} \, \frac{\sum_{i=0}^{n} i(i-1) \, p_i^{(\infty)}}{\sum_{i=0}^{n} i \, p_i^{(\infty)}} \tag{S43}$$

These summations evaluate in principle to analytical expressions in terms of hypergeometric functions. Plotting $\frac{I^{(\infty)}}{k_{\text{exc}}}$ as a function of k_{exc} reproduces the saturation curves known from the paper of Erickson *et al.*

We can calculate $g^{(2)}(0)$ from $I^{(\infty)}$ and $I^{(0)}$. It is below 1 and depends on k_{exc}. The anti-bunching is strongest at the lowest k_{exc}. In the limit of $k_{\text{exc}} \to 0$, the anti-bunching goes to

$$\lim_{k_{\text{exc}} \to 0} g^{(2)}(0) = \lim_{k_{\text{exc}} \to 0} \frac{I^{(0)}}{I^{(\infty)}} = \frac{(n-1)k_{\text{ET}}}{(n-1)k_{\text{ET}} + k_{\text{Auger}}}. \tag{S44}$$

Again, we recover the result of section S4.3 by setting $k_{\text{Auger}} = 0$.

S4.6 Emitters populated by 1-to-1 energy transfer from the host material; the host material is quenched by an Auger process; self-consistent excited population

Starting from section S4.4 and including Auger quenching, the self-consistent equation for the expected number of excited dopants becomes

$$k_{\text{exc}} \frac{k_{\text{ET}}}{np_0 k_{\text{ET}} + np_1 k_{\text{Auger}}} \, p_0 - k_{\text{Yb}} p_1 = 0. \tag{S45}$$

Here, the fraction describes competition of energy transfer to any one dopant with energy transfer to all other ground-state dopants (adding up to $np_0 k_{\text{ET}}$) and with Auger quenching by all excited-state dopants (adding up to $np_1 k_{\text{Auger}}$). As in section S4.4, the consideration of continuous variables p_0 and p_1 neglects discrete populations and fluctuations therein.

The steady-state solution is

$$p_1^{(\infty)} = \frac{k_{\text{ET}}(k_{\text{exc}} + nk_{\text{Yb}}) - \sqrt{4nk_{\text{exc}} k_{\text{Yb}} k_{\text{ET}} k_{\text{Auger}} + k_{\text{ET}}^2 (k_{\text{exc}} - nk_{\text{Yb}})^2}}{2(k_{\text{ET}} - k_{\text{Auger}}) nk_{\text{Yb}}} \tag{S46}$$

In the limit of $k_{\text{Auger}} \to 0$, this reduces to

$$p_1^{(\infty)} = \begin{cases} \dfrac{k_{\text{exc}}}{nk_{\text{Yb}}} & ; \quad k_{\text{exc}} < nk_{\text{Yb}} \\ 1 & ; \quad k_{\text{exc}} > nk_{\text{Yb}} \end{cases} \tag{S47}$$

The expected count rate is

$$I^{(\infty)} = \eta_{\text{col}} k_{\text{r}} n p_1^{(\infty)}. \tag{S48}$$

The detection of a photon signals that 1 dopant is in its ground state at $t = 0$. Just before $t = 0$, this 1 dopant was excited and thereby affected the feeding of the other $n-1$ dopants. The expected excited populations (p_0 and p_1) among the other dopants was such that

$$k_{\mathrm{exc}} \frac{k_{\mathrm{ET}}}{(n-1)p_0 k_{\mathrm{ET}} + [(n-1)p_1 + 1]\, k_{\mathrm{Auger}}}\, p_0 - k_{\mathrm{Yb}} p_1 = 0. \tag{S49}$$

This expression describes that, just before $t = 0$, energy transfer to any particular dopant (k_{ET}) competes with energy transfer to all other ground-state dopants (adding up to $(n-1)p_0 k_{\mathrm{ET}}$; excluding the 1 dopant that emitted at $t = 0$) and Auger quenching by all excited-state dopants (adding up to $[(n-1)p_1 + 1]\, k_{\mathrm{Auger}}$; surely including the 1 dopant that emitted at $t = 0$). Solving for p_1 yields the population $p_1^{(0)}$ among n_1 dopants at $t = 0$:

$$p_1^{(0)} = \frac{k_{\mathrm{Auger}} k_{\mathrm{Yb}} + k_{\mathrm{ET}}\, [k_{\mathrm{exc}} + (n-1)k_{\mathrm{Yb}}] - \sqrt{D}}{2(k_{\mathrm{ET}} - k_{\mathrm{Auger}})(n-1)k_{\mathrm{Yb}}} \tag{S50}$$

with

$$D = [k_{\mathrm{Auger}} k_{\mathrm{Yb}} + k_{\mathrm{ET}} k_{\mathrm{exc}} + k_{\mathrm{ET}}(n-1)k_{\mathrm{Yb}}]^2 + 4(k_{\mathrm{Auger}} - k_{\mathrm{ET}})k_{\mathrm{ET}} k_{\mathrm{exc}} (n-1) k_{\mathrm{Yb}}. \tag{S51}$$

We can from here calculate the expected count rate directly following the detection of a photon,

$$I^{(0)} = \eta_{\mathrm{col}} k_{\mathrm{r}} (n-1) p_1^{(0)}. \tag{S52}$$

In the limit of $k_{\mathrm{Auger}} \to 0$, this reduces to

$$I^{(0)} = \begin{cases} \eta_{\mathrm{col}} k_{\mathrm{r}} \dfrac{k_{\mathrm{exc}}}{k_{\mathrm{Yb}}} & ; \quad k_{\mathrm{exc}} < (n-1)k_{\mathrm{Yb}} \\ \eta_{\mathrm{col}} k_{\mathrm{r}} & ; \quad k_{\mathrm{exc}} > (n-1)k_{\mathrm{Yb}} \end{cases}, \tag{S53}$$

consistent with section S4.4 We can calculate the expected anti-bunching:

$$g^{(2)}(0) = \frac{I^{(0)}}{I^{(\infty)}}. \tag{S54}$$

Similar to section S4.5, this result for $g^{(2)}(0)$ approaches

$$\lim_{k_{\mathrm{exc}} \to 0} g^{(2)}(0) = \frac{(n-1)k_{\mathrm{ET}}}{(n-1)k_{\mathrm{ET}} + k_{\mathrm{Auger}}}. \tag{S55}$$

Across all possible values of k_{exc}, the maximum deviation in predicted anti-bunching magnitude between the results of sections S4.5 and S4.6 is 1.5% relative, using parameter values of Erickson *et al.* and $n = 800$. Hence, the simpler model with continuous populations p_0 and p_1 produces a very similar result to the discrete micro-state model. At smaller n, the deviation may be larger.

S4.7 Emitters populated by quantum-cutting energy transfer from the host material; the host material is quenched by an Auger process; self-consistent excited population

Starting from section S4.6, we now add the hypothesis of quantum cutting to the model. Several slightly different implementations are conceivable. We keep it simple here. First, we multiply k_{ET}

with a factor to reflect that an energy-transfer event now excites two dopants. Yet, following the arguments of Erickson *et al.* we assume here that the energy-transfer rate scales linearly with the number of ground-state Yb dopants in a nanocrystal, based on the weak Yb-doping dependence observed experimentally.[S1] The self-consistent equation for the expected number of excited dopants then is similar to section S4.6:

$$k_{\text{exc}} \frac{2k_{\text{ET}}}{np_0 k_{\text{ET}} + np_1 k_{\text{Auger}}} p_0 - k_{\text{Yb}} p_1 = 0. \tag{S56}$$

The steady solution for $p_1^{(\infty)}$ becomes

$$p_1^{(\infty)} = \left(\frac{k_{\text{exc}}}{nk_{\text{Yb}}} + \frac{1}{2} \right) \frac{k_{\text{ET}}}{k_{\text{ET}} - k_{\text{Auger}}} + \frac{\sqrt{8nk_{\text{exc}}k_{\text{Yb}}k_{\text{ET}}k_{\text{Auger}} + k_{\text{ET}}^2(2k_{\text{exc}} - nk_{\text{Yb}})^2}}{2(k_{\text{Auger}} - k_{\text{ET}})nk_{\text{Yb}}} \tag{S57}$$

which reduces in the limit of $k_{\text{Auger}} \to 0$ to

$$p_1^{(\infty)} = \begin{cases} \dfrac{2k_{\text{exc}}}{nk_{\text{Yb}}} & ; \quad 2k_{\text{exc}} < nk_{\text{Yb}} \\ 1 & ; \quad 2k_{\text{exc}} > nk_{\text{Yb}} \end{cases} \tag{S58}$$

With quantum-cutting energy transfer, the information provided by the detection of a photon is different than previously. The detected photon has:

(A) a 50% probability to be the first of a quantum-cut pair. In this scenario, we know that the emitting dopant is now in the ground state, 1 other dopant in the nanocrystal is surely in the excited state, and the other $n-2$ dopants experienced slightly different feeding just prior to $t = 0$.

(B) a 50% probability to be the second of a quantum-cut pair. In this scenario, we know that the emitting dopant is now in the ground state. The other $n-1$ dopants experience slightly different feeding just prior to $t = 0$. We assume for this calculation that the 1 dopant that emitted the first of the quantum-cut pair of photons shortly before, is part of the $n-1$ dopants. This means that it had sufficient time to equilibrate its excited-state population.

The description for scenario B is similar to section S4.6. We describe the excited-state probability of the $n-1$ dopants that did *not* just emit with

$$k_{\text{exc}} \frac{2k_{\text{ET}}}{(n-1)p_0 k_{\text{ET}} + \left[(n-1)p_1 + 1\right] k_{\text{Auger}}} p_0 - k_{\text{Yb}} p_1 = 0. \tag{S59}$$

and use the symbol $p_{1,\text{2nd}}^{(0)}$ for the solution:

$$p_{1,\text{2nd}}^{(0)} = \frac{k_{\text{Auger}}k_{\text{Yb}} + k_{\text{ET}}\left[2k_{\text{exc}} + (n-1)k_{\text{Yb}}\right] - \sqrt{D}}{2(k_{\text{ET}} - k_{\text{Auger}})(n-1)k_{\text{Yb}}} \tag{S60}$$

with

$$D = \left[k_{\text{Auger}}k_{\text{Yb}} + 2k_{\text{ET}}k_{\text{exc}} + k_{\text{ET}}(n-1)k_{\text{Yb}}\right]^2 + 8(k_{\text{Auger}} - k_{\text{ET}})k_{\text{ET}}k_{\text{exc}}(n-1)k_{\text{Yb}}. \tag{S61}$$

For scenario A, we describe the expected excited-state population $p_{1,\text{1st}}^{(0)}$ of the $n-2$ dopants

that are not part of the quantum-cutting pair just detected, with

$$k_{\text{exc}} \frac{2k_{\text{ET}}}{(n-2)p_0 k_{\text{ET}} + [(n-2)p_1 + 2]\, k_{\text{Auger}}}\, p_0 - k_{\text{Yb}} p_1 = 0. \tag{S62}$$

The solution is

$$p_{1,\text{1st}}^{(0)} = \frac{2k_{\text{Auger}} k_{\text{Yb}} + k_{\text{ET}} \left[2k_{\text{exc}} + (n-2)k_{\text{Yb}}\right] - \sqrt{D'}}{2(k_{\text{ET}} - k_{\text{Auger}})(n-2)k_{\text{Yb}}} \tag{S63}$$

with

$$D' = \left[2k_{\text{Auger}} k_{\text{Yb}} + 2k_{\text{ET}} k_{\text{exc}} + k_{\text{ET}}(n-2)k_{\text{Yb}}\right]^2 + 8(k_{\text{Auger}} - k_{\text{ET}})k_{\text{ET}} k_{\text{exc}}(n-2)k_{\text{Yb}}. \tag{S64}$$

Weighing the two scenarios 50/50, we obtain the expected count rate just after detecting a photon:

$$I^{(0)} = \eta_{\text{col}} k_{\text{r}} \left[\frac{1}{2}(n-2)\left(p_{1,\text{1st}}^{(0)} + 1\right) + \frac{1}{2}(n-1)p_{1,\text{2nd}}^{(0)}\right] \tag{S65}$$

In the limit of low excitation and large number of dopants, $g^{(2)}(0)$ becomes

$$g^{(2)}(0) \sim 1 + \frac{k_{\text{r}}}{4k_{\text{exc}}}. \tag{S66}$$

Expressing this in terms of the low-excitation limit of the steady-state count rate, $I^{(\infty)} \sim 2\eta_{\text{col}} k_{\text{exc}}$, we obtain

$$g^{(2)}(0) \sim 1 + \frac{k_{\text{r}} \eta_{\text{col}}}{2I^{(\infty)}}. \tag{S67}$$

This is the same expression as we used in Ref. Benning et al.[S3]

S4.8 Multiple nanocrystals; 1-to-1 energy transfer from the host material; the host material is quenched by an Auger process; self-consistent excited population

We adapt the model for 1-to-1 energy transfer to a situation with N nanocrystals, each doped with n dopants, as

$$k_{\text{exc}} \frac{1}{N} \frac{k_{\text{ET}}}{np_0 k_{\text{ET}} + np_1 k_{\text{Auger}}}\, p_0 - k_{\text{Yb}} p_1 = 0. \tag{S68}$$

Here, p_0 and p_1 are the excited-state populations of one particular dopant in the sample. The parameter k_{exc} is now interpreted as the excitation rate of the sample as a whole, and the factor $1/N$ describes the probability that an excitation occurs in the specific nanocrystal of the dopant considered. The solution is similar to section S4.6 [equations (S46), (S46), (S48)] but with all n replaced with Nn.

After the detection of a photon, we adapt equation (S49) to account for the probability $1/N$ that it originated from the specific nanocrystal of the dopant considered:

$$k_{\text{exc}} \frac{1}{N} \frac{k_{\text{ET}}}{(n-1/N)p_0 k_{\text{ET}} + [(n-1/N)p_1 + 1/N]\, k_{\text{Auger}}}\, p_0 - k_{\text{Yb}} p_1 = 0. \tag{S69}$$

Again, the solutions are similar to section S4.6 [equations (S50) through (S55)] but with all n replaced with Nn.

As all expressions scale only with Nn, while N and n never appear separately, we conclude that the expected (anti-)bunching depends only on the total number of dopants in the sample, $n_{\mathrm{tot}} = nN$, not on how they are distributed over nanocrystals. Note that we are still using the rate-equation approximation with continuous populations np_0 and np_1, which works well for large n (compare S4.5, S4.6) but deviations may be expected as $n \to 1$. Further deviations from the model could occur if additional excited-state pathways become relevant at high k_{exc}, such as excited-state absorption or photo-ionization of nanocrystals.

S4.9 Multiple nanocrystals; quantum-cutting energy transfer from the host material; the host material is quenched by an Auger process; self-consistent excited population

The expressions from section S4.7, for quantum-cutting energy transfer in a single nanocrystals can be adapted for N nanocrystals with each n dopants using the strategy described in section S4.8. Using the same arguments, we will find that all expressions of section S4.8 need a replacement $n \to nN$. Again, the expected (anti-)bunching depends only on the total number of dopants in the sample, $n_{\mathrm{tot}} = nN$, not only how they are distributed over nanocrystals.

S4.10 Bulk material

Sections S4.8 and S4.9 concluded that the expected (anti-)bunching depends only on the total number of dopants $n_{\mathrm{tot}} = Nn$ in a sample of N nanocrystals with each n dopants. We can thus guess that the same expressions hold for a bulk material, which is essentially one large crystal ($N = 1$) with many dopants ($n \gg 1$).

Indeed, we arrive at this conclusion if we adapt the expressions for the steady-state excited-state populations to the scenario of a bulk material. Here, we take equation (S45) as an example to illustrate the necessary adaptation. The bulk version of equation (S45) would be

$$k_{\mathrm{exc}} \frac{V_{\mathrm{ET}}}{V_{\mathrm{exc}}} \frac{k_{\mathrm{ET}}}{np_0 k_{\mathrm{ET}} + np_1 k_{\mathrm{Auger}}} p_0 - k_{\mathrm{Yb}} p_1 = 0. \tag{S70}$$

Here V_{ET} is the volume probed by a perovskite exciton, due to a combination of its finite Bohr radius and diffusion within the excited-state lifetime, and V_{exc} is the total volume of the excitation spot in the bulk material. The ratio $V_{\mathrm{ET}}/V_{\mathrm{exc}}$ describes the probability that any excitation in the bulk material is sufficiently nearby to interact with the specific dopant described with the above expression. We interpret n as the number of dopants within V_{ET}. We still assume here that $n \gg 1$, so that using continuous populations p_0 and p_1 is a reasonable approximation, as discussed above. These are the dopants that compete for the same excitation energy as the specific dopant described with the above expression. We see that $V_{\mathrm{ET}}/V_{\mathrm{exc}}$ takes the same role as $1/N$ in sections S4.8 and S4.9. All other relevant expressions similarly include factors $V_{\mathrm{ET}}/V_{\mathrm{exc}}$ wherever sections S4.8 and S4.9 would have a factor $1/N$.

Hence, the expected (anti-)bunching for a bulk material will be described with the expressions of section S4.6 (for 1-to-1 transfer) or section S4.7 (for quantum cutting) with all n replaced by $nV_{\mathrm{exc}}/V_{\mathrm{ET}} = n_{\mathrm{tot}}$. Here, n_{tot} is the total number of dopants in the excitation volume.

In Fig. 4 of the main text, we show experimental results of experiments performed at a constant count rate $I^{(\infty)}$ but with different laser spot size. Hence, the number of nanocrystals N or the total number of dopants n_{tot} is different between experiments. We plot the experimental (anti-)bunching as a function of the laser spot size σ. The size σ of the excitation spot is first estimated from the measured spot size σ_{image} on the camera. We deconvolve the imaged spot size into diffraction of excitation light and diffraction of emitted light using

$$\sigma = \sqrt{\frac{\lambda_{\text{exc}}^2}{\lambda_{\text{exc}}^2 + \lambda_{\text{em}}^2}}\sigma_{\text{image}}, \tag{S71}$$

where $\lambda_{\text{exc}} = 375$ nm is the excitation wavelength and $\lambda_{\text{em}} = 980$ nm is the emission wavelength. For the calculations, we consider that a spot with lateral size σ has a volume of

$$\begin{aligned} V_{\text{exc}}(L) &= \int_0^L \int_{-\infty}^{\infty} \int_{-\infty}^{\infty} \exp\left[-\frac{1}{2}\left(\frac{x}{\sigma}\right)^2 - \frac{1}{2}\left(\frac{y}{\sigma}\right)^2 - \frac{1}{2}\left(\frac{z}{f_z\sigma}\right)^2\right] \mathrm{d}x\,\mathrm{d}y\,\mathrm{d}z \\ &= \frac{1}{2} f_z (2\pi)^{3/2}\sigma^3 \operatorname{erf}\left(\frac{L}{\sqrt{2}f_z\sigma}\right). \end{aligned} \tag{S72}$$

Here, $f_z = 2.29$ is the theoretical focus elongation in our experiment.[S10] the lower limit of 0 for the z integration accounts for the focus being positioned at the interface between the sample and the substrate. The upper integration limit L is set to 100 nm for the nanocrystal films and to infinity for the bulk sample. In the bulk limit, $\operatorname{erf}(\infty) = 1$, such that:

$$V_{\text{exc}}^{\text{bulk}} = \frac{1}{2} f_z (2\pi)^{3/2}\sigma^3 \tag{S73}$$

This determines the total number of dopants in the excitation spot:

$$n_{\text{tot}} = \phi\rho_{\text{Yb}}V_{\text{exc}}, \tag{S74}$$

where $\rho_{\text{Yb}} = 0.228$ nm^{-3} is the Yb number density in perovskite for a doping concentration of 4%, ϕ is the volume fraction occupied by perovskite. For bulk material we use $\phi = 1$, while for nanocrystals in a nanocrystal aggregate we estimate at 50%. This makes the theoretical line for bulk material and nanocrystals slightly different.

Next, we solve for the excitation rate k_{exc} from the theoretical expressions for $I^{(\infty)}$, which depends on σ through n_{tot} and using fixed values of $\eta_{\text{col}} = 1.1\%$ and $k_{\text{r}} = 0.43$ ms^{-1}. We then plug this k_{exc}, along with the other parameter values, into the expression of $g^{(2)}(0) = I^{(0)}/I^{(\infty)}$ to obtain the expected (anti-)bunching as a function of σ.

Table S1: Overview of experimental near-infrared (NIR) photoluminescence quantum yields (PLQYs) in Yb^{3+}-doped $CsPb(Cl_{1-x}Br_x)_3$ materials reported in the literature. Entries are organized chronologically. Doping levels and PLQYs are reported as stated in the cited literature.

Material form	Size	Composition/ Yb doping level	PLQY	Measurement technique	Ref.
Nanocrystals	6.8 nm	$CsPbCl_{1.5}Br_{1.5}$:Yb^{3+}(7.1%), Ce^{3+}(2.0%)	119%	Integrating sphere	S11
Nanocrystals	6.3 nm	$CsPbCl_3$:Yb^{3+}(9.1%)	143%	Integrating sphere	S12
Nanocrystals	16 nm	$CsPbCl_3$:Yb^{3+}(0.2–7.4%)	170%	Integrating sphere	S1
Nanocrystals	10 nm	$CsPbCl_3$:Yb^{3+}(2 at.%)	128%	Integrating sphere	S13
Films	150 ± 31 nm	$CsPb(Cl_{0.35}Br_{0.65})_3$:$Yb^{3+}$	193%	Integrating sphere	S14
Nanocrystals	12.5–14.5 nm	$CsPbCl_3$:Yb^{3+}(0.6–5.7 at.%)	0.45%	Not specified	S15
Nanocrystals	15.8 ± 1.8 nm	$CsPbCl_3$:Yb^{3+}(6%)	164 ± 7%	Integrating sphere	S16
Nanocrystals	14.1 ± 3.8 nm	$CsPb(Cl_{1-x}Br_x)_3$:Yb^{3+}(5.5%, 7.7%)	200%	Integrating sphere	S17
Nanocrystals	$8^{(i)}$<br>11 $nm^{(ii)}$	$CsPbCl_3$:Yb^{3+}(0.62 at.%)$^{(i)}$<br>$CsPbCl_3$:Yb^{3+}(1.21 at.%)$^{(ii)}$	87.9%$^{(i)}$<br>58.3%$^{(ii)}$	Integrating sphere	S18
Nanocrystals	Not specified	$CsPbCl_3$:Yb^{3+}(5.4%)	130%	Integrating sphere	S19
Nanocrystals	11 nm	$CsPbCl_3$:Yb^{3+}(1.4%)	175%	Integrating sphere	S8
Thermally evaporated films	Not specified	$CsPb(Cl_{0.5}Br_{0.5})_3$:Yb^{3+}(4.7%)	183%	Integrating sphere	S2
Nanocrystals	6.5 nm$^{(i)}$<br>7–8 nm$^{(ii)}$<br>8–9 nm$^{(iii)}$	$CsPbCl_3$:Yb^{3+}(7%)$^{(i)}$<br>$CsPbCl_{1.5}Br_{1.5}$:Yb^{3+}(7%)$^{(ii)}$<br>$CsPbClBr_2$:Yb^{3+}(7%)$^{(iii)}$	85%$^{(i)}$<br>117%$^{(ii)}$<br>137%$^{(iii)}$	Integrating sphere	S20
Films	120 nm	$CsPbCl_3$:Yb^{3+}(1.0–9.1%)	62.3%	Integrating sphere	S21
Nanocrystals	10 nm	$CsPbCl_3$:Yb^{3+}($<$ 1 at.%)	60%	Integrating sphere	S22
Nanocrystals	13.4 ± 1.9 nm	$CsPbCl_3$:Yb^{3+}(2.7%)	150%	Integrating sphere	S23
Nanocrystals	11.7–13.9 nm	$CsPbCl_{1.5}Br_{1.5}$:Yb^{3+}(5%–30%)	89.2%	Integrating sphere	S24
Nanocrystals	15 nm	$CsPbCl_3$:Yb^{3+}(6.2%)	129%	Not specified	S25
Nanocrystals	8 nm	$CsPbCl_3$:Yb^{3+}(8.4%), Ce^{3+}(8.2% or 3%)	175%	Not specified	S26
Nanocrystals	12 nm	$CsPbCl_3$:Yb^{3+}(2.3–2.5%), Mn^{2+}(1.2–1.5%)	103%	Integrating sphere	S27
Single crystals/ bulk/ powders	Not specified	$CsPbCl_3$:Yb^{3+}(9 at.%)	75.8%	Integrating sphere	S28
Single crystals	3 × 2 × 2 mm	$CsPbCl_3$:Yb^{3+}(2%)	20%	Integrating sphere	S29
Films	Not specified	$CsPbCl_3$:Yb^{3+}(50%)	133%	Integrating sphere	S30
Nanocrystals	15 nm	$CsPbCl_3$:Yb^{3+}(7 at.%)	110%	Integrating sphere	S31
Nanocrystals	15 nm	$CsPbCl_3$:Yb^{3+}(4.5 at.%)	127%	Not specified	S32
Nanocrystals	13.6 ± 2.3 nm	$CsPbCl_3$:Yb^{3+}(1.2%)	130%	Relative dye method	S33
Nanocrystals	100–300 nm	$CsPbCl_3$:Yb^{3+}(6.8%)$^{(i)}$<br>$CsPbClBr_2$:Yb^{3+}(6.8%)$^{(ii)}$	102%$^{(i)}$<br>128%$^{(ii)}$	Integrating sphere	S34
Nanocrystals	8.2 ± 0.1 nm	$CsPbCl_3$:Yb^{3+}(8.4%), La^{3+}(8.7%)	168%	Integrating sphere	S35
Nanocrystals	8.6 ± 0.8 – 8.9 ± 1.1 nm	$CsPb(Cl_{0.9}Br_{0.1})_3$:Yb^{3+}(4.5%)	177%	Integrating sphere	S36
Nanocrystals	13.18 nm	$CsPbCl_3$:Yb^{3+}(15–16%)	63%	Not specified	S37
Films	250–467 nm, grain 33–48 nm	$CsPbCl_3$:Yb^{3+}(25%)	33.94%	Integrating sphere	S38
Single crystals/ bulk/ powders	5 × 5 × 3 mm	$CsPbCl_3$:$Yb^{3+(i)}$<br>$CsPbCl_{1.5}Br_{1.5}$:$Yb^{3+(ii)}$<br>$CsPbClBr_2$:$Yb^{3+(iii)}$	136.5%$^{(i)}$<br>148.7%$^{(ii)}$<br>108.5%$^{(iii)}$	Integrating sphere	S39
Nanocrystals	11–12 nm	$CsPbCl_3$:Yb^{3+}(2.12 at.%)	138.6%	Integrating sphere	S40

Material form	Size	Composition/ Yb doping level	PLQY	Measurement technique	Ref.
Nanocrystals	16 nm	$CsPb(Cl_{1-x}Br_x)_3$:Yb^{3+} (4%)	126%	Integrating sphere	S41
Films	105 nm	$CsPbCl_3$:Yb^{3+}(0.2–0.8 M precursor)	27.1–72.4%	Integrating sphere	S42
Thermally evaporated films	–	$CsPbCl_3$:Yb^{3+}(2–10%)	70%	Integrating sphere	S43
Nanocrystals	13.9–14.7 nm	$CsPbCl_3$:Yb^{3+}(15%)	63%	Not specified	S44
Mechanosynthesized powders/ films	35–85 nm	$CsPbCl_3$:Yb^{3+}(5.1%)	162%	Integrating sphere	S45
Nanocrystals	9.4 ± 1.5 nm	$CsPb(Cl_{1-x}Br_x)_3$:Yb^{3+} (30.2%)	139%	Integrating sphere	S46
Single crystals/ bulk/ powders	Not specified	$CsPbCl_3$:Yb^{3+}(2%)	86%	Integrating sphere/ radiometer	S47
Nanocrystals	10.3–12.5 nm	$CsPbCl_xBr_{3-x}$:Yb^{3+}	68.1%	Not specified	S48
mechanosynthesized powders$^{(i)}$/ films$^{(ii)}$	Not specified	$CsPb(Cl_{1-x}Br_x)_3$:Yb^{3+} (0.5–50%)	114%$^{(i)}$ 107%$^{(ii)}$	Integrating sphere	S49
Nanocrystals	10 nm	$CsPbCl_3$:Yb^{3+}, Ru^{3+}	152%	Integrating sphere	S50
Nanocrystals	9 nm	$CsPbCl_3$:Yb^{3+}(3.1 at.%)	170–192%	Integrating sphere	S51
Nanocrystals	5.67 ± 2.18 nm	$CsPbCl_3$:Yb^{3+}	100%	Integrating sphere	S52
Nanocrystals	10 nm	$CsPbCl_3$:Yb^{3+}(< 10%)	16.7–24.3%	Integrating sphere	S53
Nanocrystals	16.1 ± 0.5 nm	$CsPbClBr_2$:Yb^{3+}	78%	Integrating sphere	S54
Solution-processed films	Not specified	$CsPbCl_3$:Yb^{3+}	143%	Integrating sphere	S55
Powders	Grain 1–6 μm	$CsPbCl_3$:Yb^{3+}(10%), Rb^+(5–40%)	78%	Integrating sphere	S56
Thermally evaporated films	150 nm	$CsPbCl_3$:Yb^{3+}(10 at.%)	96.8%	Integrating sphere	S57
Single crystals	30 × 40 mm	$CsPbCl_3$:Yb^{3+}(2%$^{(i)}$, 5%, 10%)	71.20$^{(i)}$%	Integrating sphere	S58
Quantum dot polycrystalline film hybrids	9.8 nm	$CsPbCl_{1.5}Br_{1.5}$:Yb^{3+} in $CsPbCl_{1.5}Br_{1.5}$	159%	Integrating sphere	S59
Mechanosynthesized powder	352 nm	$CsPbCl_3$:Yb^{3+}(5%)	78%	Integrating sphere	S60
Thermally evaporated films	120 nm	$CsPbCl_3$:Yb^{3+}(8 at.%)	60.5% 91.9%	fluorescence spectrometer	S61
Nanocrystals	10 nm	$CsPbCl_3$:Yb^{3+}(1.6 at.%)$^{(i)}$ $CsPbCl_3$:Yb^{3+}(3.4 at.%) $^{(ii)}$	120%$^{(i)}$ 166%$^{(ii)}$	Visible/NIR detector response-corrected photon-counting	S62
Nanocrystals	3.89 nm	$CsPbCl_3$:Yb^{3+}(10.96 mol.%)	146%	Integrating sphere	S63
Nanocrystals	15.1 ± 2.6 nm	$CsPbCl_3$:Yb^{3+}(3.3–3.5 at.%)	155.2%	Integrating sphere	S64
Chiral quantum dots	14 nm	$CsPbCl_3$:Yb^{3+}	157.2%	fluorescence spectrophotometer	S65
Nanocrystals	10 nm ± 1.7$^{(i)}$ 9.0 nm ± 1.1$^{(ii)}$ 7.5 nm ± 0.8$^{(iii)}$ 6.7 nm ± 0.6$^{(iiii)}$	$CsPbCl_3$:Yb^{3+}(3.5 at.%)$^{(i)}$ $CsPbCl_3$:Yb^{3+}(6 at.%)$^{(ii)}$ $CsPbCl_3$:Yb^{3+}(8 at.%)$^{(iii)}$ $CsPbCl_3$:Yb^{3+}(9.5 at.%)$^{(iiii)}$	120%$^{(i)}$ 130%$^{(ii)}$ 137%$^{(iii)}$ 170%$^{(iiii)}$	Integrating sphere	S66

References

[S1] Milstein, T. J., Kroupa, D. M. & Gamelin, D. R. Picosecond quantum cutting generates photoluminescence quantum yields over 100% in ytterbium-doped $CsPbCl_3$ nanocrystals. *Nano Lett.* **18**, 3792–3799 (2018).

[S2] Crane, M. J. *et al.* Single-source vapor deposition of quantum-cutting Yb^{3+}: $CsPb(Cl_{1-x}Br_x)_3$ and other complex metal-halide perovskites. *ACS Appl. Energy Mater.* **2**, 4560–4565 (2019).

[S3] Benning, V. R. M. *et al.* Photon statistics as a tool to (dis) prove cooperative energy transfer quantum cutting in near-infrared emitting materials. *ACS Energy Lett.* **10**, 4620–4626 (2025).

[S4] Polyanskiy, M. N. Refractiveindex. info database of optical constants. *Sci. Data* **11**, 94 (2024).

[S5] Ermolaev, G. *et al.* Giant and tunable excitonic optical anisotropy in single-crystal halide perovskites. *Nano Lett.* **23**, 2570–2577 (2023).

[S6] McGinty, S. M., Kapala, M. K. & Niedziela, R. F. Mid-infrared complex refractive indices for oleic acid and optical properties of model oleic acid/water aerosols. *Phys. Chem. Chem. Phys.* **11**, 7998–8004 (2009).

[S7] Karaveli, S. & Zia, R. Spectral tuning by selective enhancement of electric and magnetic dipole emission. *Phys. Rev. Lett.* **106**, 193004 (2011).

[S8] Erickson, C. S., Crane, M. J., Milstein, T. J. & Gamelin, D. R. Photoluminescence saturation in quantum-cutting Yb^{3+}-doped $CsPb(Cl_{1-x}Br_x)_3$ perovskite nanocrystals: Implications for solar downconversion. *J. Phys. Chem. C* **123**, 12474–12484 (2019).

[S9] Sokolova, A. V. *et al.* Stoichiometry control in dual-band emitting Yb^{3+}-doped $CsPbCl_xBr_{3-x}$ perovskite nanocrystals. *J. Phys. Chem. C* **126**, 20550–20557 (2022).

[S10] Beyer, H. *Handbuch der Mikroskopie* (VEB Verlag Technik, Berlin, 1985), 2 edn.

[S11] Zhou, D. *et al.* Cerium and ytterbium codoped halide perovskite quantum dots: a novel and efficient downconverter for improving the performance of silicon solar cells. *Adv. Mater.* **29**, 1704149 (2017).

[S12] Pan, G. *et al.* Doping lanthanide into perovskite nanocrystals: highly improved and expanded optical properties. *Nano Lett.* **17**, 8005–8011 (2017).

[S13] Zhang, X. *et al.* Yb^{3+} and Yb^{3+}/Er^{3+} doping for near-infrared emission and improved stability of $CsPbCl_3$ nanocrystals. *J. Mater. Chem. C* **6**, 10101–10105 (2018).

[S14] Kroupa, D. M., Roh, J. Y., Milstein, T. J., Creutz, S. E. & Gamelin, D. R. Quantum-cutting ytterbium-doped $CsPb(Cl_{1-x}Br_x)_3$ perovskite thin films with photoluminescence quantum yields over 190%. *ACS Energy Lett.* **3**, 2390–2395 (2018).

[S15] Zhou, L. *et al.* Dual-emission and two charge-transfer states in ytterbium-doped cesium lead halide perovskite solid nanocrystals. *J. Phys. Chem. C* **122**, 26825–26834 (2018).

[S16] Luo, X., Ding, T., Liu, X., Liu, Y. & Wu, K. Quantum-cutting luminescent solar concentrators using ytterbium-doped perovskite nanocrystals. *Nano Lett.* **19**, 338–341 (2018).

[S17] Milstein, T. J. *et al.* Anion exchange and the quantum-cutting energy threshold in ytterbium-doped CsPb $(Cl_{1-x}Br_x)_3$ perovskite nanocrystals. *Nano Lett.* **19**, 1931–1937 (2019).

[S18] Ma, J.-P. *et al.* Insights into the local structure of dopants, doping efficiency, and luminescence properties of lanthanide-doped $CsPbCl_3$ perovskite nanocrystals. *J. Mater. Chem. C* **7**, 3037–3048 (2019).

[S19] Cohen, T. A. *et al.* Quantum-cutting Yb^{3+}-doped perovskite nanocrystals for monolithic bilayer luminescent solar concentrators. *J. Mater. Chem. A* **7**, 9279–9288 (2019).

[S20] Zhou, D. *et al.* Impact of host composition, codoping, or tridoping on quantum-cutting emission of ytterbium in halide perovskite quantum dots and solar cell applications. *Nano Lett.* **19**, 6904–6913 (2019).

[S21] Ishii, A. & Miyasaka, T. Sensitized Yb^{3+} luminescence in $CsPbCl_3$ film for highly efficient near-infrared light-emitting diodes. *Adv. Sci.* **7**, 1903142 (2020).

[S22] Lesage, A., van der Laan, M., Gomez, L. & Gregorkiewicz, T. Substitutional doping of Yb^{3+} in $CsPbBr_xCl_{3-x}$ nanocrystals. *J. Phys. Chem. C* **124**, 6413–6417 (2020).

[S23] Zeng, M. *et al.* Boosting the Er^{3+} 1.5 μm luminescence in $CsPbCl_3$ perovskite nanocrystals for photonic devices operating at telecommunication wavelengths. *ACS Appl. Nano Mater.* **3**, 4699–4707 (2020).

[S24] Zhao, S., Zhang, Y. & Zang, Z. Room-temperature doping of ytterbium into efficient near-infrared emission $CsPbBr_{1.5}Cl_{1.5}$ perovskite quantum dots. *Chem. Commun.* **56**, 5811–5814 (2020).

[S25] Zhu, Y. *et al.* Effective infrared emission of erbium ions doped inorganic lead halide perovskite quantum dots by sensitization of ytterbium ions. *J. Alloys Compd.* **835**, 155390 (2020).

[S26] Ding, N. *et al.* Extremely efficient quantum-cutting Cr^{3+}, Ce^{3+}, Yb^{3+} tridoped perovskite quantum dots for highly enhancing the ultraviolet response of silicon photodetectors with external quantum efficiency exceeding 70%. *Nano Energy* **78**, 105278 (2020).

[S27] Cai, T. *et al.* Mn^{2+}/Yb^{3+} codoped $CsPbCl_3$ perovskite nanocrystals with triple-wavelength emission for luminescent solar concentrators. *Adv. Sci.* **7**, 2001317 (2020).

[S28] Stefanski, M., Ptak, M., Sieradzki, A. & Strek, W. Optical characterization of Yb^{3+}: $CsPbCl_3$ perovskite powder. *Chem. Eng. J.* **408**, 127347 (2021).

[S29] Roh, J. Y. D. *et al.* Yb^{3+} speciation and energy-transfer dynamics in quantum-cutting Yb^{3+}-doped $CsPbCl_3$ perovskite nanocrystals and single crystals. *Phys. Rev. Mater.* **4**, 105405 (2020).

[S30] Ishii, A. & Miyasaka, T. Quantum cutting-induced near-infrared luminescence of Yb^{3+} and Er^{3+} in a layer structured perovskite film. *J. Chem. Phys.* **153** (2020).

[S31] Huang, H. *et al.* Ytterbium-doped $CsPbCl_3$ quantum cutters for near-infrared light-emitting diodes. *ACS Appl. Mater. Interfaces* **13**, 34561–34571 (2021).

[S32] Xu, K., Chen, D., Huang, D. & Zhu, H. Thermally stable emission from Yb^{3+}-doped $CsPbCl_3$ nanocrystals. *J. Lumin.* **240**, 118464 (2021).

[S33] Chang, W. J., Irgen-Gioro, S., Padgaonkar, S., Lopez-Arteaga, R. & Weiss, E. A. Photoredox-mediated sensitization of lanthanide dopants by perovskite nanocrystals. *J. Phys. Chem. C* **125**, 25634–25642 (2021).

[S34] Sun, R. *et al.* In situ preparation of two-dimensional ytterbium ions doped all-inorganic perovskite nanosheets for high-performance visual dual-bands photodetectors. *Nano Energy* **93**, 106815 (2022).

[S35] Yang, G. *et al.* Efficient quantum cutting of lanthanum and ytterbium ions co-doped perovskite quantum dots towards improving the ultraviolet response of silicon-based photodetectors. *J. Alloys Compd.* **921**, 166097 (2022).

[S36] Shen, X. *et al.* Near-infrared leds based on quantum cutting-activated electroluminescence of ytterbium ions. *Nano Lett.* **23**, 82–90 (2022).

[S37] Liu, X. *et al.* Broad-spectrum germanium photodetector based on the ytterbium-doped perovskite nanocrystal downshifting effect. *ACS Appl. Opt. Mater.* **1**, 507–512 (2022).

[S38] Becerril, L. *et al.* Water as the only solvent for $CsPbCl_3$ and NIR-emitting $CsPbCl_3$:Yb^{3+} films by antisolvent-enhanced crystallization in aerosol-assisted chemical vapor deposition. *Adv. Mater. Technol.* **8**, 2201890 (2023).

[S39] Zi, L. *et al.* X-Ray quantum cutting scintillator based on $CsPbCl_xBr_{3-x}$: Yb^{3+} single crystals. *Laser Photonics Rev.* **17**, 2200852 (2023).

[S40] Li, D. & Chen, G. Near-infrared photoluminescence from ytterbium-and erbium-codoped $CsPbCl_3$ perovskite quantum dots with negative thermal quenching. *J. Phys. Chem. Lett.* **14**, 2837–2844 (2023).

[S41] Yu, Y.-J. *et al.* Efficient near-infrared electroluminescence from lanthanide-doped perovskite quantum cutters. *Angew. Chem. Int. Ed.* **62**, e202302005 (2023).

[S42] Li, H. *et al.* Realization of 1.54-μm light-emitting diodes based on Er^{3+}/Yb^{3+} co-doped $cspbcl_3$ films. *Adv. Mater.* **35**, 2300118 (2023).

[S43] Cleveland, I. J. *et al.* Physical vapor deposition of Yb-doped $CsPbCl_3$ thin films for quantum cutting. *Phys. Rev. Mater.* **7**, 065404 (2023).

[S44] Ye, Z. *et al.* 980 nm near-infrared light-emitting diode using all-inorganic perovskite nanocrystals doped with ytterbium ions. *Tsinghua Sci. Technol.* **29**, 207–215 (2023).

[S45] Streckaitė, S. *et al.* Facile synthesis of ytterbium doped cesium lead halide perovskite powder. *J. Mater. Chem. C* **11**, 15463–15472 (2023).

[S46] Pan, J.-L. *et al.* Polarity-mediated antisolvent control enables efficient lanthanide-based near-infrared perovskite leds. *Nano Lett.* **24**, 2765–2772 (2024).

[S47] Demkiv, T. *et al.* Effect of Yb doping on the optical and photoelectric properties of $CsPbCl_3$ single crystals. *Opt. Mater.: X* **22**, 100303 (2024).

[S48] Tepliakov, N. V. *et al.* Trap-mediated sensitization governs near-infrared emission from Yb^{3+}-doped mixed-halide $CsPbCl_xBr_{3-x}$ perovskite nanocrystals. *Nano Lett.* **24**, 3347–3354 (2024).

[S49] Streckaitė, S. *et al.* The limiting factors of quantum-cutting efficiency of ytterbium-doped lead halide perovskites: dimeric and monomeric ytterbium species. *J. Mater. Chem. C* **12**, 11995–12003 (2024).

[S50] Wang, Y. *et al.* Efficient large-area quantum cutting photoconversion films for silicon solar cells on photovoltaic glass using knife coating. *J. Phys. Chem. Lett.* **15**, 7236–7243 (2024).

[S51] Jing, Y. *et al.* Stable and highly emissive infrared yb-doped perovskite quantum cutters engineered by machine learning. *Adv. Mater.* **36**, 2405973 (2024).

[S52] Fang, X. *et al.* Photo-induced synthesis of ytterbium and manganese-doped $CsPbCl_3$ nanocrystals for visible to near-infrared photoluminescence with negative thermal quenching. *Adv. Sci.* **12**, 2408927 (2025).

[S53] Van de Voorde, M., Hudry, D., Busko, D., Richards, B. S. & Saive, R. $CsPbCl_3$:Yb^{3+} nanocrystals: Adverse effects of colloidally stable ytterbium-rich reaction by-products on luminescent down-conversion performance. *Opt. Mater.: X* **26**, 100407 (2025).

[S54] Fang, X. *et al.* Plasmon-enhanced luminescence in ytterbium-doped halide perovskite nanocrystals for versatile applications. *J. Alloys Compd.* **1032**, 181105 (2025).

[S55] Yang, H. *et al.* Efficient and stable ytterbium-doped $CsPbCl_3$ near-infrared light-emitting diodes via additive-assisted crystallization. *Laser Photonics Rev.* **19**, e00757 (2025).

[S56] Jagiełowicz, W., Bondzior, B., Rajfur, K., Ptak, M. & Stefanski, M. Effect of Rb^+ co-doping on the optical properties in VIS and NIR emitting $CsPbCl_3$:Yb^{3+} perovskite powder. *Dalton Trans.* **54**, 14191–14198 (2025).

[S57] Wang, S. *et al.* Exciton localization engineering in thermally evaporated Yb-doped $CsPbCl_3$ near-infrared light-emitting diodes. *Adv. Mater.* **38**, e13853 (2026).

[S58] Zhu, Y. *et al.* Near-infrared emitting rare-earth doped perovskite scintillator for X-Ray flexible imaging. *Laser Photonics Rev.* **20**, e02227 (2026).

[S59] Li, W. *et al.* 986/1540 nm quantum cutting-based perovskite near-infrared light-emitting diodes: Overcoming bottlenecks via a quantum dot-matrix hybrid strategy. *Small Methods* **10**, e02163 (2026).

[S60] Rubio, T. I. & Avalos, C. E. Defect tolerant quantum cutting in mechanosynthesized ytterbium-doped cesium lead chloride perovskites. *Chem. Mater.* (2026).

[S61] Zhang, S. *et al.* Crystal facet engineering in thermally evaporated ytterbium-doped perovskite near-infrared light-emitting diodes. *ACS Nano* (2026).

[S62] Mukherjee, P. & Sarma, D. Mechanistic insights into quantum-cutting in Yb^{3+}-doped $CsPbCl_3$ nanocrystals. *Small* **22**, e14834 (2026).

[S63] Wang, S. *et al.* Facile synthesis of stable Yb^{3+}-doped perovskite nanocrystals in mesoporous silica for near-infrared emission. *ACS Applied Materials & Interfaces* **18**, 37908–37917 (2026).

[S64] Yang, Y. *et al.* Robust quantum cutting via halide-bearing ligand passivation and gradient halide reconstruction for ultrabroadband ultraviolet-to-near-infrared photodetection and imaging. *ACS nano* (2026).

[S65] Liu, W. *et al.* Chiral quantum-cutting. *Journal of the American Chemical Society* (2026).

[S66] Cheng, Y. *et al.* Site-dominant quantum-cutting nanocrystals enable silicon photovoltaics exceeding 100% external quantum efficiency. *Joule* (2026).
